# High Peptide Dose Vaccination Promotes the Early Selection of Tumor Antigen-Specific CD8 T-Cells of Enhanced Functional Competence

**DOI:** 10.3389/fimmu.2019.03016

**Published:** 2020-01-08

**Authors:** Laura Carretero-Iglesia, Barbara Couturaud, Petra Baumgaertner, Julien Schmidt, Hélène Maby-El Hajjami, Daniel E. Speiser, Michael Hebeisen, Nathalie Rufer

**Affiliations:** Department of Oncology UNIL CHUV, Lausanne University Hospital, University of Lausanne, Lausanne, Switzerland

**Keywords:** melanoma, vaccination, CD8 T-cells, peptide and CpG-B doses, NTAmers, TCR-pMHC binding avidity, CD8 binding dependency, functional avidity

## Abstract

CD8 T-cell response efficiency critically depends on the TCR binding strength to peptide-MHC, i.e., the TCR binding avidity. A current challenge in onco-immunology lies in the evaluation of vaccine protocols selecting for tumor-specific T-cells of highest avidity, offering maximal immune protection against tumor cells and clinical benefit. Here, we investigated the impact of peptide and CpG/adjuvant doses on the quality of vaccine-induced CD8 T-cells in relation to binding avidity and functional responses in treated melanoma patients. Using TCR-pMHC binding avidity measurements combined to phenotype and functional assays, we performed a comprehensive study on representative tumor antigen-specific CD8 T-cell clones (*n* = 454) from seven patients vaccinated with different doses of Melan-A/ELA peptide (0.1 mg vs. 0.5 mg) and CpG-B adjuvant (1–1.3 mg vs. 2.6 mg). Vaccination with high peptide dose favored the early and strong *in vivo* expansion and differentiation of Melan-A-specific CD8 T-cells. Consistently, T-cell clones generated from those patients showed increased TCR binding avidity (i.e., slow off-rates and CD8 binding independency) readily after 4 monthly vaccine injections (4v). In contrast, the use of low peptide or high CpG-B doses required 8 monthly vaccine injections (8v) for the enrichment of anti-tumor T-cells with high TCR binding avidity and low CD8 binding dependency. Importantly, the CD8 binding-independent vaccine-induced CD8 T-cells displayed enhanced functional avidity, reaching a plateau of maximal function. Thus, T-cell functional potency following peptide/CpG/IFA vaccination may not be further improved beyond a certain TCR binding avidity limit. Our results also indicate that while high peptide dose vaccination induced the early selection of Melan-A-specific CD8 T-cells of increased functional competence, continued serial vaccinations also promoted such high-avidity T-cells. Overall, the systematic assessment of T-cell binding avidity may contribute to optimize vaccine design for improving clinical efficacy.

## Introduction

Recent advances in onco-immunology have enabled new progress in the development of cancer vaccines. Therapeutic cancer vaccination aims at generating a strong and persistent anti-tumor immune response, targeting tumor cells, and overcoming immunosuppression in the tumor microenvironment. Specifically, therapeutic vaccines should mobilize high frequencies of powerful effector and memory T lymphocytes to fight against cancer in a highly specific way. Personalized vaccines amplifying T-cell responses against mutated neoantigens have shown to efficiently elicit poly-specific and poly-functional T-cells using synthetic peptides (1, 2) or whole-tumor cell lysate (3). Beside the prioritization of tumor antigens, vaccine formulation represents another essential aspect of therapeutic vaccination (4). Antigenic peptides and adjuvant CpG B-ODN emulsified in the mineral oil-based Incomplete Freund Adjuvant (IFA) is currently one of the most potent vaccine formulations for promoting the expansion of *ex vivo*-detectable tumor antigen-specific CD8 T-cell responses in melanoma patients (5, 6). Such vaccine can generate functionally competent T-cells *in vivo* (7, 8) and correlate with favorable clinical outcome (9). Therefore, there is a strong rational to further exploit these powerful vaccines in combination with other effective agents, especially with immune checkpoint inhibitory antibodies.

Many observations support the importance of considering not only quantitative (i.e., magnitude of response) but also qualitative (i.e., functional avidity) determinants of the T-cell response to predict the clinical efficacy of therapeutic vaccination (10, 11). In that regard, increasing the functional avidity of T-cells was found to be tightly associated with efficient viral clearance (12–16) and enhanced tumor growth control (17–20). Functional avidity of T-cells has also been related to the antigen dose used for vaccination, with increasing doses negatively correlating to reduced T-cell avidity (13). Importantly, whereas functional avidity of CD8 T-cells has been shown to be highly dependent on the antigen dose during the *in vitro* culture expansion (13, 17), only few reports have observed a relationship between vaccine antigen dose and functional avidity *in vivo* (21, 22). Indeed, most attempts to prime high avidity CD8 T-cells by *in vivo* vaccination have failed, mainly because it remains difficult to induce effective T-cell responses through vaccination with low antigen doses [reviewed in (23)]. Recently, by combining a novel potent adjuvant with low-dose immunization, Billeskov et al. (24) found that low antigen dose selectively primed CD4 T-cells of higher functional avidity and protective efficacy in mice. By contrast, CD8 T-cell functional avidity remained unrelated to the vaccine dose (24). In cancer patients, we previously reported that vaccination with low peptide dose induced tumor antigen-specific CD8 T-cells of enhanced cytotoxicity (i.e., maximal T-cell responses at saturating antigen concentrations), but there was no difference in their functional avidity (i.e., specific T-cell responses when exposed to increasing antigen concentrations) (25). Hence, the precise impact of peptide dose on both functional and binding avidity of T-cells still remains to be determined in well-defined human anti-tumor vaccination settings.

The functional avidity is primarily controlled by the strength by which the T-cell receptor (TCR) binds to cognate peptide-MHC (pMHC). In fact, the TCR binding avidity represents a critical parameter for tumor/self antigen-specific CD8 T-cell responses, usually mediated by TCRs of relatively low avidity. Consequently, there is a large body of evidence revealing that enhanced TCR-pMHC binding avidity correlates with augmented T-cell functionality (26–30) as well as *in vivo* improved tumor growth control in cancer patients (31, 32). Using fluorescent reversible NTAmers, we recently showed that the TCR-pMHC binding avidity accurately predicted T-cell functional potency of anti-cancer and virus-specific CD8 T-cell responses (33). Moreover, we performed a complete characterization of TCR-pMHC avidity of tumor-specific CD8 T-cells induced by peptide-based vaccination of melanoma patients and found differences in TCR-pMHC binding avidity depending on the type of Melan-A^MART−1^_26−35_ peptide used for vaccination. Precisely, vaccination with a low dose of native Melan-A_26−35_ peptide together with IFA and CpG-B induced CD8 T-cells with higher TCR binding avidity and stronger tumor reactivity compared to vaccination with the analog Melan-A_26−35_ A27L peptide (8, 34). Together, the NTAmer approach offers a strong biometric, by which the quality of tumor antigen-specific CD8 T-cell responses can be directly evaluated and graded in order to better characterize their impact on the efficacy of cancer-based therapies.

Here, we investigated the effect of Melan-A peptide and adjuvant CpG-B doses on the binding and functional avidity of vaccine-induced antigen-specific CD8 T-cells from melanoma patients after multiple monthly vaccine injections. We found that high peptide dose vaccine (i.e., 0.5 mg) promoted the early selection (after 4 vaccine injections) of Melan-A-specific T-cells of increased TCR binding avidity (i.e., slow off-rates and low CD8 binding dependency), correlating with enhanced functional avidity. This contrasted to the T-cell responses obtained after low peptide (i.e., 0.1 mg) or high CpG-B (2.6 mg vs. 1–1.3 mg) dose vaccines requiring instead higher numbers of vaccinations (i.e., 8 vaccines). We further observed a positive correlation between TCR binding avidity and functional avidity. However, above a given TCR-pMHC binding avidity limit, T-cell function could not be further augmented, revealing a plateau of maximal T-cell functional competence, corresponding to optimal functional avidity. Altogether, TCR binding avidity is a well-suited biometric to evaluate and guide the rational development of therapeutic cancer-based vaccines.

## Patients and Methods

### Patients and Vaccination Protocol

HLA-A2-positive patients with histologically proven metastatic (stage III/IV) melanoma of the skin were included in phase I prospective trials [ClinicalTrials.gov; Identifiers: NCT00112229 and NCT00112242; (5, 35)]. Primary endpoints were safety and tolerability, as well as detailed measurements of tumor antigen-specific CD8 T-cell responses over time. Eligible patients received monthly vaccinations injected subcutaneously with the analog Melan-A^MART−1^_26−35_ (A27L) peptide (ELAGIGILTV, ELA) at 0.1 mg (low dose, 10 patients) or 0.5 mg (high dose, 5 patients). LAU1015 received 20 injections of low dose of native Melan-A^MART−1^_26−35_ peptide (EAAGIGILTV, EAA) before being included in the cohort of patients vaccinated with high analog/ELA peptide dose. Vaccine emulsions were prepared with peptide, IFA (Montanide ISA-51; Seppic), and with either low dose (1 or 1.3 mg) or high dose (2 or 2.6 mg) of CpG-B 7909/PF-3512676 (Pfizer and Coley Pharmaceutical Group) as described elsewhere (5). The patient's characteristics including previous treatments are shown in Supplementary Tables 1, 2. Vaccination was well-tolerated with side effects mostly being inflammatory granuloma at subcutaneous injection sites (data not shown). Peripheral blood mononuclear cells (PBMCs) were isolated using Ficoll-Hypaque (Pharmacia), cryopreserved in 10% DMSO (Sigma-Aldrich) and stored in liquid nitrogen until further use.

### Direct *ex vivo* Immuno-Monitoring and Generation of Melan-A-Specific CD8 T-Cell Clones

After thawing, PBMCs were positively enriched using anti-CD8-coated magnetic microbeads (Miltenyi Biotec). CD8-positive T-cell fractions were stained in PBS, 0.2% BSA, and 5 mM EDTA with PE-labeled HLA-A^*^ 0201 multimers loaded with analog Melan-A_26−35_ (A27L) (Peptide and Tetramer Core Facility, Ludwig Cancer Research, UNIL CHUV, Lausanne, Switzerland) at 4°C for 45 min, followed then with appropriate antibodies at 4°C for 30 min, as described in the Supplemental Materials and Methods section. Samples for direct *ex vivo* immune-monitoring were acquired on a LSRII cytometer (BD Biosciences) and analyzed using FlowJo 9.7.6 software (TreeStar, Inc). For the generation of directly *ex vivo* sorted tumor-specific T-cell clones, antibody-stained Melan-A-specific CD8 T-cells were first sorted as early differentiated effector-memory (EM28^pos^, CD45RA^neg^CCR7^neg^CD28^pos^) and late differentiated effector-memory (EM28^neg^, CD45RA^neg^CCR7^neg^CD28^neg^) subsets, using a FACSAria (BD Biosciences) flow cytometer, as described in Supplemental Materials and Methods section. *Ex vivo* sorted multimer^pos^ CD8 T-cell subsets were then cloned by limiting dilution in Terasaki plates and expanded by stimulation with 150 U/ml recombinant human IL-2 (GlaxoSmithKline), 1 μg/ml PHA (Remel) and 1 × 10^6^/ml irradiated allogeneic PBMCs (30-Gy) as feeder cells. Once established, vaccine-induced Melan-A-specific CD8 T-cell clones were maintained in culture and periodically expanded (every 18–20 days) by stimulation with PHA, recombinant human IL-2 and irradiated feeder cells. T-cell clones were cryopreserved in 10% DMSO (Sigma-Aldrich) and stored in liquid nitrogen until further use.

### NTAmer Staining and Dissociation Kinetic Measurements

NTAmers were synthetized by the Peptide and Tetramer Core Facility, Ludwig Cancer Research, UNIL CHUV (Lausanne, Switzerland) as described previously (36). Dually labeled NTAmers are composed of streptavidin-phycoerythrin (SA-PE) complexed with biotinylated peptides and non-covalently bound to His-tagged HLA-A^*^0201 monomers containing Cy5-labeled β2m (36) and were used for dissociation kinetic measurements as described previously (34, 37). Briefly, individual vaccine-induced Melan-A-specific CD8 T-cell clones were stained for 45 min at 4°C in PBS, 0.2% BSA, and 5 mM EDTA with Melan-A-specific NTAmers, in which HLA-A^*^0201 molecules were either loaded with the native (EAA) or the analog (ELA) Melan-A_26−35_ peptide. When indicated, we also used CD8 binding-deficient HLA-A^*^0201 monomers bearing the D227K/T228A mutations in the HLA-α3 domain (38) and loaded with the analog ELA peptide. NTAmer staining was assessed at 4°C on a SORP-LSR II flow cytometer (BD Biosciences). Following 25 s of baseline acquisition, 100 mM of imidazole was added allowing the rapid dissociation of the SA-PE-NTA_4_ scaffold and monomeric Cy5 fluorescence was measured during the following 5 min. Data were analyzed using the kinetic module of FlowJo software (v.9.7.6, Tree Star) and modeled (1-phase exponential decay) using Prism software (v.6, GraphPad).

### Surface Marker Expression/Modulation Assay and Intracellular Staining

Vaccine-induced Melan-A-specific CD8 T-cell clones were incubated in the absence or presence of HLA-A^*^0201 unlabeled multimers loaded with native Melan-A_26−35_ (1 μg/ml) peptide. After 24 h of stimulation, cells were stained in PBS, 0.2% BSA, 5 mM EDTA, and 0.2% NaN_3_ for PE anti-panTCRαβ, Pacific Blue anti-CD8α and PE anti-CD8β (from Beckman Coulter), and BV421 anti-PD-1 (Biolegend) at 4°C for 30 min and acquired on a LSRII flow cytometer (BD Biosciences). The intracellular content of granzyme B and perforin was measured under unstimulated (resting) and stimulated (unlabeled native Melan-A_26−35_ multimers as described above) conditions upon cellular fixation in PBS 1% formaldehyde, 2% glucose, and 5 mM NaN_3_ for 20 min at room temperature, then stained in PBS, 0.2% BSA, 5 mM EDTA, and 0.1% saponin (Sigma-Aldrich) with FITC anti-granzyme B or APC anti-perforin (Biolegend). Vivid Aqua (Life Technologies) was used to discriminate live/dead cells. The level of expression of each marker (geometric mean fluorescence intensity [gMFI]) was analyzed using FlowJo software (v.10.0.7).

### CD107a Degranulation and Intracellular Cytokine Staining

HLA-A^*^0201-positive TAP-deficient T2 cells were pulsed 1 h at 37°C with serial dilutions of the native Melan-A_26−35_ peptide (from 10^−4^ to 10^−11^ M), washed, and incubated with vaccine-induced tumor antigen-specific CD8 T-cell clones at an E/T ratio of 1:2 for 6 h in the presence of FITC anti-CD107a antibody (BD Pharmingen) and brefeldin A (10 μg/ml, Sigma-Aldrich). Cells were then stained in PBS, 0.2% BSA, and 5 mM EDTA with Pacific Blue anti-CD8α (Beckman Coulter) at 4°C for 30 min, fixed in PBS 1% formaldehyde, 2% glucose, and 5 mM NaN_3_ for 20 min at room temperature, and finally stained in PBS, 0.2% BSA, 5 mM EDTA, and 0.1% saponin (Sigma-Aldrich) with PE-Cy7 anti-IFNγ and Alexa700 antibodies anti-TNFα (BD Pharmingen) for 30 min at 4°C before acquisition on a LSRII (BD Biosciences) flow cytometer. Percentages of CD107a/cytokine-positive T-cells were analyzed using FlowJo software (v.10.0.7, Tree Star). EC_50_ and Bmax values were derived by dose-response curve analysis (log[agonist] vs. response) using Prism software. Non-CD107a-degranulation or non-cytokine clones were defined as displaying a maximal response <25% and for which an EC_50_ value could not accurately be determined. These clones were not included in the statistical analyses.

### Calcium Mobilization Assay

Calcium mobilization assay was performed as described previously (37). Briefly, vaccine-induced Melan-A-specific CD8 T-cells were loaded with 2 μM Indo 1-AM (Sigma-Aldrich) for 1 h, washed and resuspended at a concentration of 10^6^ cells in pre-warmed RPMI containing 2% of FCS. Baseline signal was recorded for 30 s before 1 μg/ml analog HLA-A2/Melan-A_26−35_-specific multimers were added and intracellular Ca^2+^ flux was assessed for 5 min under UV excitation and constant temperature (37°C) using a thermostat device on an LSR II SORP (BD Biosciences) flow cytometer. Indo-1 (violet)/Indo-1 (blue) 405/425 nm emission ratio was analyzed by the FlowJo kinetics module software (v.9.7.6, Tree Star).

### Chromium Release Cytolytic Assay

HLA-A^*^0201-positive TAP-deficient T2 cells, HLA-A, B-deficient C1R cells expressing the wild-type HLA-A2 (C1R WT) or CD8 binding-deficient HLA-A2 (HLA-A^*^0201^D227K/T228A^, C1R CD8-null) molecules were used as target cells (39). Chromium release cytolytic assays were performed as previously described (20). Briefly, ^51^Cr-labeled HLA-A2-positive T2 cells, or C1R WT and C1R CD8-null cells were pulsed with serial dilutions of native Melan-A_26−35_ peptide (from 10^−6^ to 10^−13^ M) and incubated with Melan-A-specific CD8 T-cell clones at an E/T ratio of 10:1 for 4 h at 37°C. Percentage of specific lysis was calculated as 100 × (experimental – spontaneous release)/(total – spontaneous release). EC_50_ and B_max_ values were obtained by dose-response curve analysis (log[agonist] vs. response) using Prism software.

### Statistics

Data were analyzed using Prism software (v.6, GraphPad) by nonparametric Mann-Whitney, Wilcoxon matched-pairs signed rank test and Spearman's correlation. The associated *P*-values as well as numbers of experiments and sample sizes are indicated throughout the manuscript.

## Results

### Patients and Vaccinations

In this study, fifteen HLA-A2-positive patients with metastatic melanoma received monthly vaccinations s.c. with low (0.1 mg, *n* = 10) or high (0.5 mg, *n* = 5) doses of the analog Melan-A^MART−1^_26−35_ A27L peptide (referred hereafter as ELA), supplemented with IFA and CpG-B. Low peptide dose-vaccinated patients received progressive increasing doses over time (i) of 0.5 to 1 mg and to 2 mg of CpG-B (protocol LUD00-018) or (ii) of 1.3 mg up to 2.6 mg of CpG-B (protocol LUD01-003) as described in Supplementary Figure 1. Besides Melan-A peptide, all patients received additional peptides in their vaccine formulations, for targeting multiple tumor antigens (Supplementary Table 1). We found that these other peptides did not significantly affect the specific CD8 T-cell responses to Melan-A (40). Our findings support the notion that T-cells with different specificities usually respond relatively independently of each other, provided that immunization is done with vaccines in which the different peptides are not covalently linked to each other. The high immunodominance of A2/Melan-A specific responses may further enforce the independence of this particular CD8 T-cell response from T-cells specific for other epitopes.

### Early *in vivo* Expansion and Differentiation of Melan-A-Specific CD8 T-Cells After Vaccination With High Peptide Dose

**Figure 1:**
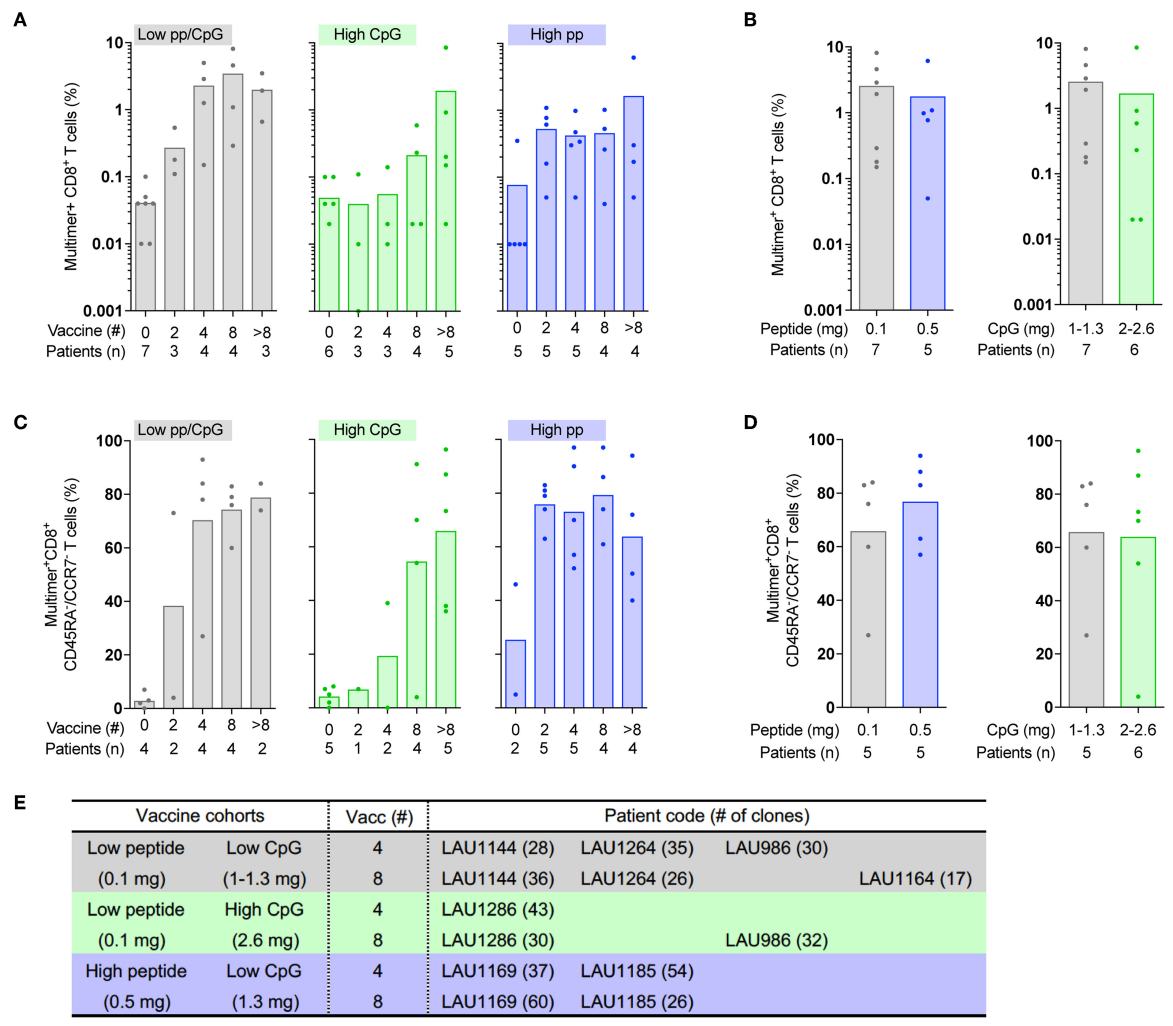
Direct *ex vivo* frequencies and cell differentiation of circulating Melan-A-specific CD8 T-cells following vaccination with increased peptide or CpG doses. Blood samples of melanoma patients receiving monthly therapeutic vaccinations with different doses of Melan-A/ELA peptide or CpG with IFA were analyzed before the start of vaccination (0) and after the indicated numbers of vaccinations (2, 4, 8, and >8) **(A,C)** or at the response peak **(B,D)**. **(A–E)** There were three cohorts of patients, according to the peptide or CpG dose [low peptide (0.1 mg)/low CpG (1–1.3 mg), gray bars; low peptide (0.1 mg)/high CpG (2–2.6 mg), green bars; high peptide (0.5 mg)/low CpG (1.3 mg), blue bars]. Each dot represents a single patient. **(A)** Quantification of Melan-A/ELA-specific CD8 T-cell frequencies. **(B)** Comparison of the maximum Melan-A/ELA-specific CD8 T-cell frequencies reached during the study between low vs. high peptide dose (left panel) and low vs. high CpG dose (right panel). **(C)** Multimer^pos^ CD8 T-cells were characterized directly *ex vivo* by flow cytometry for CCR7 and CD45RA expression. Proportion of differentiated effector-memory (EM, CD45RA^neg^CCDR7^neg^) CD8 T-cells according to the type of vaccination. **(D)** The proportion of differentiated EM Melan-A/ELA-specific CD8 T-cells at the peak of response was compared between low vs. high peptide dose (left panel) and low vs. high CpG dose (right panel). **(E)** Selection of the seven melanoma patients included in this study, according to vaccine cohort and number of vaccine injections (4v vs. 8v). The number of clones generated for each patient and time-point of vaccination is indicated.

The aim of this study was to determine the impact of peptide and adjuvant doses on the generation of anti-tumor T-cell responses in relation to TCR binding avidity and functional potency. We first addressed the question whether different vaccination protocols had an influence on the expansion and differentiation of Melan-A-specific CD8 T-cells (Figure 1). Three patient cohorts were studied, according to the injected peptide and CpG-B doses; (i) low ELA peptide (0.1 mg) and low CpG-B (1–1.3 mg) (defined as low peptide/CpG thereafter; gray bars), (ii) low ELA peptide (0.1 mg) and high CpG-B (2.6 mg) (defined as high CpG; green bars), and (iii) high ELA peptide (0.5 mg) and low CpG-B (1.3 mg) (defined as high peptide; blue bars). Strong expansion of Melan-A-specific CD8 T-cells was detected after vaccination in all three cohorts, when compared to frequencies found prior vaccination, as monitored by staining with fluorescent multimers directly *ex vivo* (Figure 1A). We further found a progressive increase in frequencies of tumor antigen-specific T-cells along vaccination, yet the expansion kinetics were variable, depending on the cohort of patients. Whereas, frequencies increased up to 10-fold between 2 and 4 vaccinations in patients receiving the low peptide/CpG dose vaccine, there was a delay in patients vaccinated with high CpG dose, reaching similar frequencies only after 8 vaccines. In the high peptide dose cohort, induction of Melan-A-specific T-cells was maximal already after 2 vaccine injections with no further increase in frequencies in four out of the five studied patients (Figure 1A, Supplementary Figure 1). However, no differences were observed between low vs. high peptide and low vs. high CpG doses when we considered the maximum of Melan-A-specific CD8 T-cell frequencies reached during the study (Figure 1B). Of note, four out of the six patients, mostly included in the high CpG dose cohort, initially received 4 vaccine injections of the native Melan-A peptide before being further treated with the analog ELA peptide (Supplementary Table 1). The delay in the *ex vivo* Melan-A-specific T cell expansion found in this cohort may in fact be explained by our previous observations (8, 35) showing lower natural peptide-induced T cell frequencies when compared with vaccination with the analog peptide.

In agreement with our recent report (8), *ex vivo* Melan-A-specific CD8 T-cells from the three cohorts typically displayed a naïve-like phenotype before the start of vaccination and progressively differentiated into effector-memory phenotype (EM; CD45RA^neg^/CCR7^neg^) along vaccination, as based on the expression of CD45RA and CCR7 (Figure 1C, Supplementary Figure 2). There also was a progressive loss of CD28 expression within the multimer-specific CD8 T-cells during vaccination, despite the large variations found among some patients (Supplementary Figure 1). The establishment of EM T-cells following vaccination was again different between the patient cohorts and paralleled their expansion profile (Figure 1A). Patients vaccinated with low peptide/CpG or high CpG required larger numbers of vaccinations to reach their levels of maximally differentiated EM T-cells (Figure 1C). Conversely, vaccine-induced T-cells from patients receiving the high peptide dose mostly displayed an EM phenotype after only two vaccine injections, which was maintained with further vaccinations. Finally, the proportion of EM T-cells was highly similar between all cohorts of patients at the peak of response (Figure 1D).

Overall, the three types of vaccination (low peptide/CpG, high CpG and high peptide) could elicit a strong *in vivo* effect with the increase in frequency and differentiation of Melan-A-specific CD8 T-cells and their maintenance over time following vaccination. However, the high peptide (0.5 mg) dose vaccine induced a more rapid expansion and effector-memory differentiation than low peptide (0.1 mg) or high CpG (2.6 mg). These observations suggest a specific impact of high peptide dose occurring early after the initiation of treatment, while patients receiving low peptide/CpG or high CpG doses needed additional injections to reach the same vaccine-induced effect.

### High Peptide Dose Favors the Early Selection of Vaccine-Induced CD8 T-Cells With Increased TCR Binding Avidity

**Figure 2:**
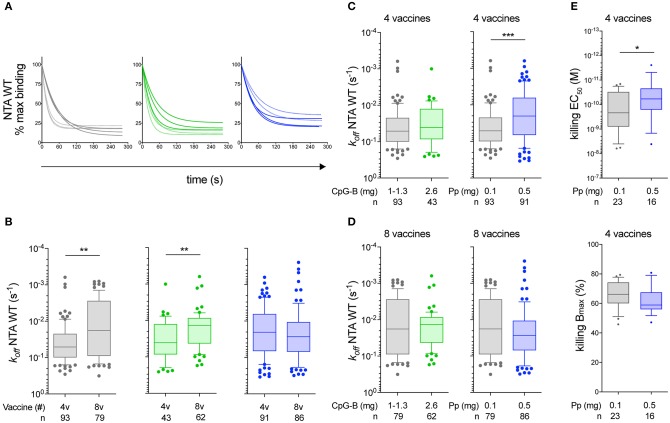
Monomeric TCR/CD8-pMHC dissociation rates (k_off_) of Melan-A-specific CD8 T-cell clones. **(A)** Representative wild-type NTAmer-based dissociation curves obtained from Melan-A-specific CD8 T-cell clones *ex vivo* derived from patients vaccinated with either low peptide/CpG (gray), high CpG (green), or high peptide (blue) dose protocol, after 4 (dashed curves) or 8 (plain curves) vaccinations. **(B)** k_off_ values categorized according to the vaccination cohort and the number of vaccine injections (4v vs. 8v). **(C,D)** Direct comparison of the dissociation rates (k_off_) between low peptide/CpG and high CpG (left panel) or high peptide (right panel) dose cohort after 4 vaccine **(C)** or 8 vaccine **(D)** injections. **(E)** EC_50_ (top panel) and B_max_ (bottom panel) values from cytolytic assays performed with chromium-labeled Melan-A-negative T2 target cells loaded with graded concentrations of native Melan-A peptide. Direct comparison between low peptide/CpG (in gray) or high peptide (in blue) dose cohort after 4 vaccine injections. **(A–E)** Data are representative of pooled tumor antigen-specific T-cell clones derived from EM28^pos^ and EM28^neg^ and are depicted as box (25th and 75th percentiles) and whisker (10th and 90th percentiles) with the middle line indicating the median. Number of clones *n* and *P*-values by Mann-Whitney *U* test are indicated; ^*^P < 0.05, ^**^P < 0.01, and ^***^P < 0.001. Pp, peptide.

TCR-ligand dissociation rate stands out as a robust biomarker to directly evaluate and quantify the potency of an immunotherapy intervention (e.g., the type of peptide used for vaccination) in melanoma patients (33, 34). To explore the impact of TCR/CD8-binding avidity for pMHC on different vaccine-induced T-cell responses according to peptide and CpG doses, we generated large representative libraries of Melan-A-specific CD8 T-cell clones (*n* = 454) derived from effector-memory CD28^pos^ (early-differentiated EM28^pos^) or CD28^neg^ (late-differentiated EM28^neg^) cells by direct *ex vivo* sorting and cloning from seven melanoma patients (Figure 1E, Supplementary Figure 3). These patients were illustrative of the three vaccination cohorts and whenever possible were followed over time from 4 to 8 vaccine injections (Supplementary Figure 2). Monomeric TCR/CD8-pMHC dissociation kinetics (i.e., off-rates or *k*_off_) were assessed on vaccine-induced CD8 T-cell clones by the two-color reversible NTAmers (36, 37). Representative dissociation curves found for the tumor antigen-specific CD8 T-cell clones induced after each vaccination protocols are depicted in Figure 2A.

For the low peptide/CpG and high CpG dose cohorts of patients, we observed a significant enrichment of slower dissociation rates (i.e., low k_off_) within the T-cell clones only after 8 vaccine compared to 4 vaccine injections (*P* < 0.01) (Figure 2B). This contrasted to the T-cell clones generated from the high peptide dose cohort showing no significant differences between the two time-points (Figure 2B). This was mainly related to the observation that Melan-A-specific T-cell clones derived from the high peptide dose cohort displayed slower off-rates (i.e., low k_off_) than those obtained from the low peptide/CpG dose protocol, readily after 4 vaccine injections (*P* < 0.001) (Figure 2C). Consequently, similar k_off_-rates were found within the vaccine-induced T-cell clones from the different CpG doses, but the prevalence of slower dissociation rates increased with the number of vaccinations (4v vs. 8v) (Figures 2C,D). Comparable data were obtained when the tumor antigen-specific T-cell clones were subdivided according to their early-differentiated EM28^pos^ origin (Supplementary Figure 4).

These results indicate that high peptide dose vaccine favors the early selection (after 4v) of tumor antigen-specific CD8 T-cells expressing TCRs with increased binding avidity (i.e., slower off-rates) compared to low peptide/CpG or high CpG dose. To determine whether this translates into enhanced functional potency, we further compared the killing capacity of T-cell clones derived from low peptide/CpG vs. high peptide dose cohorts after 4 vaccine injections (Figure 2E). The functional avidity (EC_50_, defined as the peptide concentration producing half-maximal response) was found increased in Melan-A-specific CD8 T-cell clones from the high peptide dose vaccine, compared to those generated from the low peptide/CpG dose protocol (*P* < 0.05). This correlated to their slower TCR off-rates (Figure 2C). Finally, the maximal biological responses (B_max_, defined as the maximally reached function at saturating peptide doses) were highly comparable. This is in line with our recent report (33) showing that NTAmer-determined TCR binding avidity was associated to the functional avidity (EC_50_), but not to maximal killing function (B_max_).

### High Peptide Dose Promotes the Early Enrichment of Vaccine-Induced T-Cells Expressing CD8 Binding-Independent TCRs

Beside its role in cell signaling (41, 42), the CD8 coreceptor enhances antigen recognition and T-cell activation by stabilizing and strengthening the interactions between TCR and pMHC molecules (37, 43, 44). Here, we aimed at assessing the contribution of CD8 on the overall TCR/CD8-pMHC binding avidity of vaccine-induced CD8 T-cells obtained from the three patient cohorts. Therefore, we used CD8 binding-deficient NTAmers (i.e., CD8-null NTAmers), containing the HLA-A^*^0201^D227K/T228A^ mutations in the MHC-α3 domain and thus preventing CD8 binding to pMHC (37, 38). Moreover, the proportion of differentiated effector-memory EM28^neg^ Melan-A-specific T-cell subset was highly variable among the treated melanoma patients (Supplementary Figure 1). To avoid any potential bias, all subsequent analyses were performed on T-cell clones derived from the early-differentiated EM28^pos^ cells, mostly composed of highly heterogenous TCR clonotype repertoires, including the co-dominant clonotypes found in the EM28^neg^ subset (45).

**Figure 3:**
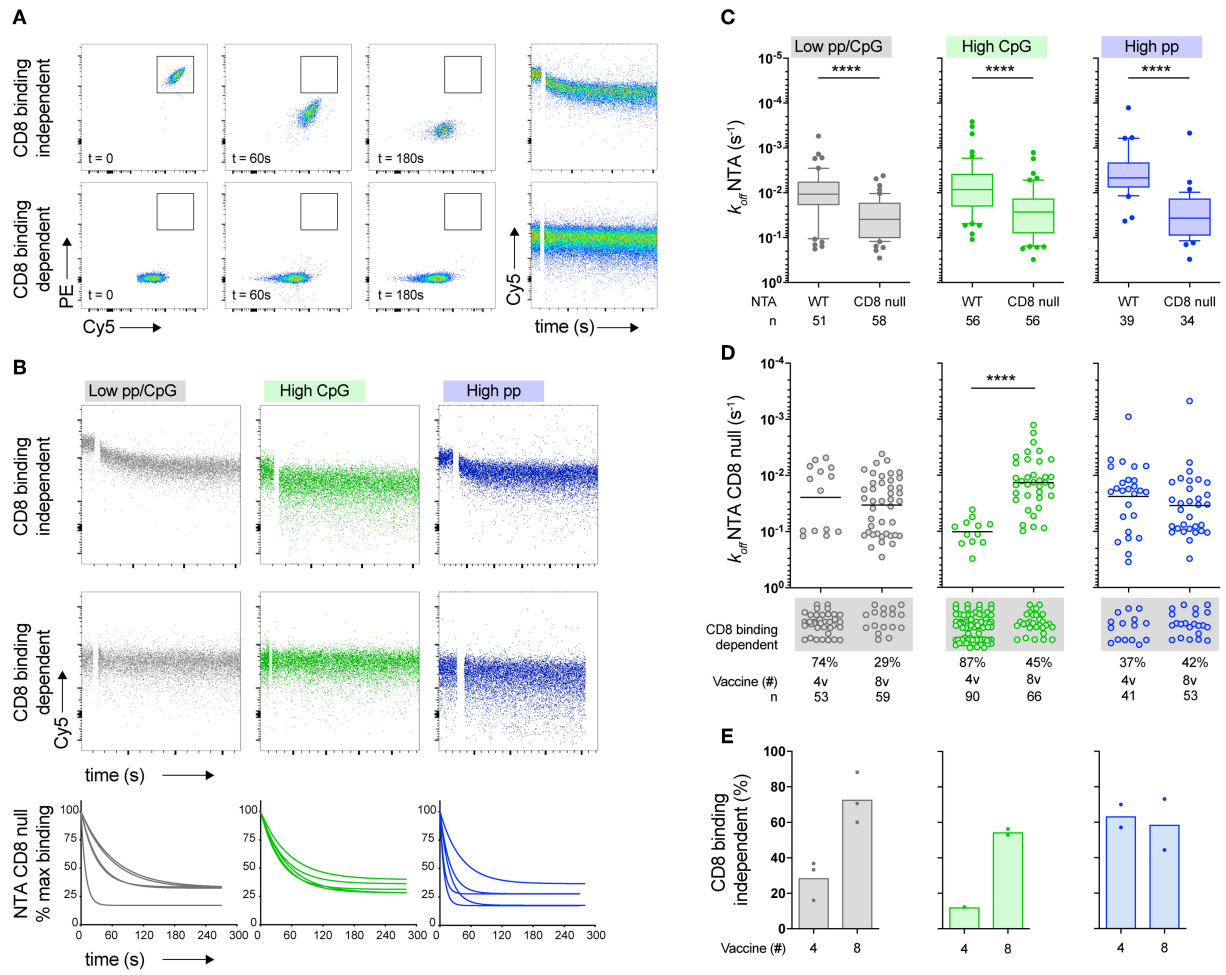
Contribution of CD8 binding to TCR/CD8-pMHC dissociation rates (k_off_) of Melan-A-specific CD8 T-cell clones. **(A)** Representative FACS-based staining dot plots at different time-points and dissociation curves from two tumor antigen-specific CD8 T-cell clones obtained after labeling with CD8 binding-deficient NTAmers. Imidazole was added after 25 s of baseline recording. The upper panels represent a T-cell clone capable to bind to CD8-null NTAmers (defined as CD8 binding-independent) with monomeric TCR-pMHC dissociation followed in the Cy5 channel during 270 s. The lower panels represent a T-cell clone that is unable to bind to CD8-null NTAmers (defined as CD8 binding-dependent). **(B)** Representative FACS-based dissociation curves obtained from Melan-A-specific CD8 T-cell clones derived from patients vaccinated with either low peptide/CpG (gray), high CpG (green), or high peptide (blue) dose protocol. CD8 binding-independent (upper panels) and CD8 binding-dependent (low panels) tumor antigen-specific T-cell clones are depicted. CD8-null NTAmer-based dissociation curves for CD8 binding-independent clones are shown (*n* = 5 for each vaccine cohort). **(C)** Direct comparison of the dissociation rates (k_off_) between wild-type and CD8-null NTAmers for each vaccine cohort. Data are representative of pooled EM28^pos^ tumor antigen-specific T-cell clones (*n* = 294) and are depicted as box (25th and 75th percentiles) and whisker (10th and 90th percentiles) with the middle line indicating the median. *P*-values by Wilcoxon matched-pairs signed rank test; ^****^P < 0.0001. **(D)** CD8-null NTAmer-based dissociation rates (k_off_) categorized according to the vaccination cohort and the number of vaccine injections (4v vs. 8v). CD8 binding-dependent Melan-A-specific EM28^pos^ T-cell clones from which k_off_ could not be accurately calculated are depicted below the graphs. Total number of clones *n* and *P*-values by Mann-Whitney *U* test are indicated; ^****^P < 0.0001. **(E)** Frequency of EM28^pos^ tumor antigen-specific T-cell clones of low CD8 binding dependency (i.e., CD8 binding-independent) according to the vaccination cohort and the number of vaccine injections (4v vs. 8v).

CD8-null NTAmer-based dissociation measurements allowed identifying two distinct subgroups of vaccine-induced CD8 T-cell clones. The first subgroup was still able to bind to the CD8-null NTAmers and monomeric dissociation rates could be accurately estimated (Figure 3A, top panels). Since Melan-A-specific T-cell clones falling in this category did not require CD8 attachment during TCR-pMHC binding dissociation assays, we defined them as CD8 binding-independent clones (i.e., low CD8 binding dependency). Conversely, the second subgroup was characterized by T-cell clones that failed to recognize the CD8-null NTAmers (Figure 3A, bottom panels). These clones were further categorized as CD8 binding-dependent, because CD8 binding was in this case essential to the TCR-pMHC interactions. Both subgroups (i.e., CD8 binding-dependent and -independent clones) were present in the three patient cohorts (Figure 3B). We next compared each cohort of vaccine-induced T-cells and found that CD8-null NTAmer-based off-rates were in all cohorts significantly faster (i.e., high k_off_) than those obtained with the wild-type NTAmers (*P* < 0.0001; Figure 3C). With the exception of the high CpG dose vaccine, no significant differences were observed in the dissociation rates between 4 to 8 vaccines when analyzing T-cell clones generated from the low peptide/CpG or high peptide dose cohorts (Figure 3D). Interestingly, we observed a progressive increase in the frequency of CD8 binding-independent T-cells of low peptide/CpG and high CpG dose protocols from 4 to 8 vaccinations. This contrasted to the high peptide dose-induced T-cell clones, largely composed of CD8 binding-independent cells already after 4 vaccine injections (Figure 3E).

Collectively, these data indicate a rapid and determining effect of the high peptide dose vaccine on the selection of tumor antigen-specific CD8 T-cells bearing TCRs of low CD8 binding dependency (i.e., NTAmer-based CD8 binding-independent), already after 4 vaccine injections. In comparison, low peptide/CpG and high CpG dose vaccines also promoted the enrichment of Melan-A-specific CD8 binding-independent T-cells, but this required continued serial vaccinations (>4v).

### Vaccine-Induced T-Cell Clones Bearing CD8 Binding-Independent TCRs Display Enhanced Binding Avidity and PD-1 Expression, Irrespectively of the Vaccine Protocol

**Figure 4:**
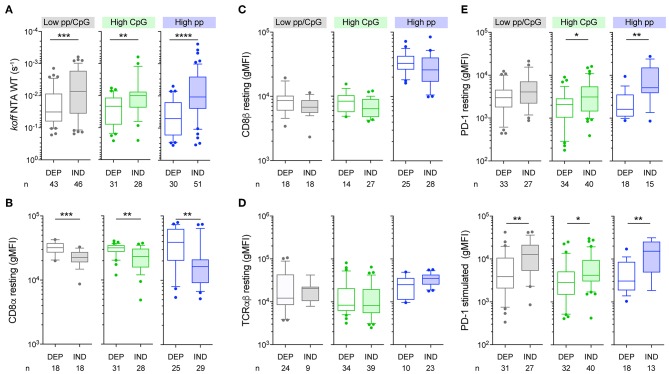
TCRαβ, CD8αβ, and PD-1 expression on CD8 binding-dependent and binding-independent Melan-A-specific CD8 T-cell clones. **(A)** Wild-type NTAmer-based dissociation rates (k_off_) of EM28^pos^ tumor antigen-specific T-cell clones categorized according to the vaccination cohort and their CD8 binding dependency. **(B–D)** Quantification of the expression levels (gMFI) under resting conditions of CD8α **(B)**, CD8β **(C)**, and TCRαβ **(D)** according to the vaccination cohort and the CD8 binding dependency status. **(E)** Quantification of PD-1 expression levels (gMFI) for CD8 binding-dependent and -independent tumor antigen-specific T-cell clones under resting conditions (upper panels) and following 24 h of stimulation with Melan-A-specific multimers (lower panels). **(A–E)** Data are depicted as box (25th and 75th percentiles) and whisker (10th and 90th percentiles) with the middle line indicating the median. Number of clones *n* and *P*-values by Mann-Whitney *U* test are indicated; ^*^P < 0.05, ^**^P < 0.01, ^***^P < 0.001, and ^****^P < 0.0001. Data are representative of 1–5 independent experiments. DEP, CD8 binding-dependent T-cell clones; IND, CD8 binding-independent T-cell clones.

We next investigated whether tumor antigen-specific T-cells expressing CD8 binding-independent TCRs showed a differential capacity to bind to pMHC complexes via the TCR and CD8 coreceptor than CD8 binding-dependent T-cell clones. To do so, we measured TCR/CD8-pMHC off-rates (by using wild-type NTAmers) as well as TCRαβ and CD8αβ surface expression on T-cell clones derived from the early-differentiated EM28^pos^ cells, isolated from the three patient cohorts. Melan-A-specific T-cell clones defined as CD8 binding-independent TCRs [based on CD8-null NTAmer dissociation assays (Figure 3)] displayed significantly slower dissociation rates (i.e., low k_off_) than the CD8 binding-dependent ones (Figure 4A). This was observed for each vaccine cohort. CD8 binding-independent T-cell clones also showed reduced CD8α (Figure 4B) and a slight trend for decreased CD8β expression (Figure 4C), while expressing comparable surface levels of TCRαβ (Figure 4D). We consistently observed greater expression of PD-1 in all cohorts of vaccine-induced T-cells bearing CD8 binding-independent TCRs, when cells were assessed in a resting state or following 24 h stimulation with Melan-A peptide (Figure 4E). This was in line with the increased levels of the CD69 activation marker expressed by those T-cells (Supplementary Figure 5). Altogether, these data indicate that vaccine-induced T-cells expressing TCRs of low CD8 binding dependency (i.e., NTAmer-based CD8 binding-independent) showed enhanced TCR binding avidity and PD-1 expression. Importantly, these features were shared among the CD8 binding-independent T-cell clones, irrespective of peptide and CpG-B dose used for vaccination. Finally, our findings are in agreement with previous studies reporting that PD-1 levels positively correlate to the strength of TCR-pMHC interactions (46), possibly compensating for T-cell over-activation (47).

### Vaccine-Induced T-Cell Clones Expressing CD8 Binding-Independent TCRs Show Enhanced Functional Avidity

**Figure 5:**
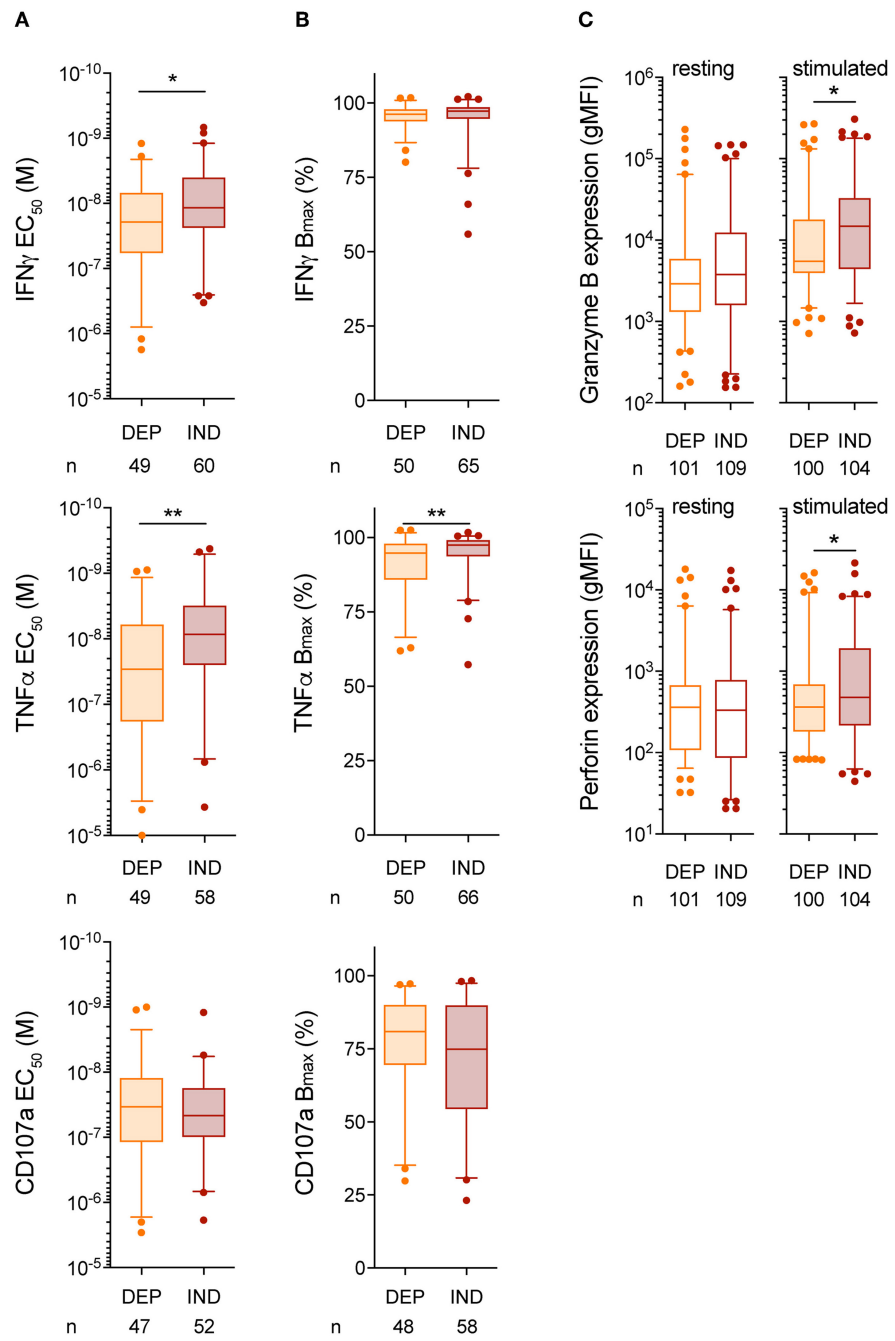
Functional competence of CD8 binding-dependent and -independent Melan-A-specific CD8 T-cell clones. **(A,B)** EC_50_ **(A)** and B_max_ **(B)** values from IFNγ, TNFα, and CD107a production performed in 6 h co-culture assays using Melan-A-negative T2 target cells pulsed with graded concentration of native Melan-A peptide. **(C)** Quantification of granzyme B and perforin intracellular expression for CD8 binding-dependent and -independent tumor antigen-specific T-cell clones. Clones were tested under resting or following 24 h of stimulation with native Melan-A-specific multimers. **(A–C)** Data are representative of pooled EM28^pos^ Melan-A-specific CD8 T-cell clones obtained from the three vaccine cohorts and categorized as CD8 binding-dependent or -independent. Data are depicted as box (25th and 75th percentiles) and whisker (5th and 95th percentiles) with the middle line indicating the median. Number of clones *n* and *P*-values by Mann-Whitney *U* test are indicated; ^*^P < 0.05 and ^**^P < 0.01. Data are representative of 2–4 independent experiments. DEP, CD8 binding-dependent T-cell clones; IND, CD8 binding-independent T-cell clones.

Previous reports have shown that the degree of CD8 dependency for T-cell activation and function inversely correlated to the TCR-pMHC binding avidity (37, 48). To address this point, CD107a degranulation and cytokine production based on peptide titration assays were performed on a representative selection of vaccine-induced EM28^pos^ T-cell clones, previously identified as either CD8 binding-dependent or binding-independent based on CD8-null NTAmer dissociation assays (Figure 3). We observed that T-cell clones of low CD8 binding dependency (i.e., CD8 binding-independent) displayed increased functional avidity compared to the IFNγ and TNFα production obtained from the CD8 binding-dependent ones (Figure 5A). These differences were more marked for the vaccine-induced T-cell clones derived from the high peptide dose cohort (Supplementary Figure 6). Again, maximal biological responses were similar between CD8 binding-dependent and binding-independent T-cell clones (Figure 5B, Supplementary Figure 6). Moreover, CD8 binding-independent T-cell clones were able to express more granzyme B and perforin than their CD8 binding-dependent counterparts upon specific stimulation (Figure 5C). Note that functional assays depend on the T-cell's activation state and can be influenced by interexperimental variabilities, while this is not the case for the TCR-pMHC off-rate measurements (33). Consequently, a large comparative study of the functional avidity between vaccine-induced CD8 binding-dependent and -independent T-cell clones, across the three patient cohorts and the two time-points (4v vs. 8v) could not be performed. Lastly, since PD-1 was found upregulated in CD8 binding-independent T-cell clones, we also tested whether the functional competence of these T-cells could be recovered upon incubation with anti-PD-1 mAb (nivolumab). PD-1 blockade had an impact on maximal function, but not on functional avidity (EC_50_) (Supplementary Figure 7).

### Vaccine-Induced T-Cell Clones Bearing CD8 Binding-Independent TCRs Reach a Plateau of Maximal Functional Avidity

**Figure 6:**
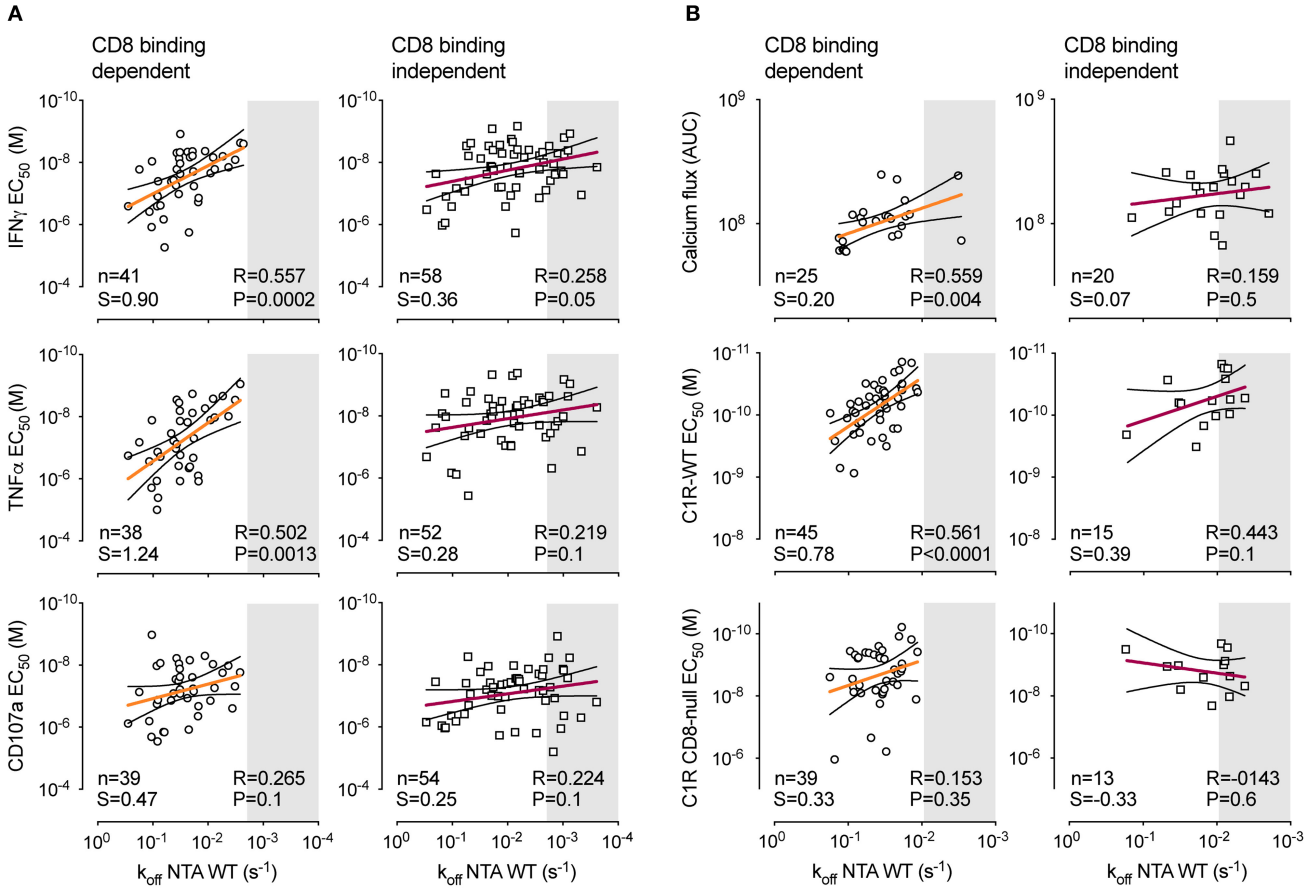
Relationship between TCR dissociation rates and functional avidity of CD8 binding-dependent and -independent Melan-A-specific CD8 T-cell clones. **(A)** Correlations between NTAmer-based TCR dissociation rates (k_off_, x-axis) and EC_50_ values obtained from IFNγ and TNFα production as well as CD107a degranulation (y-axis). **(B)** Correlations between NTAmer-based TCR dissociation rates (k_off_, x-axis) and EC_50_ values obtained from Ca^2+^ flux (AUC, area under the curve) and killing assays using wild-type (WT) or CD8 binding-deficient (CD8-null) C1R cells (y-axis). **(A,B)** Data are representative of pooled EM28^pos^ and EM28^neg^ tumor antigen-specific CD8 T-cell clones categorized as CD8 binding-dependent or binding-independent. Each symbol represents an individual clone with CD8 binding-dependent (opened circles) and CD8 binding-independent (opened scares) T-cell clones. The number of tested clones, the best-fit slope (S) value of the linear regression and the Spearman's correlation (two-tailed, α = 0.05) coefficient *R* and *P*-values are indicated. Color-coded and black lines are indicative of regression fitting and 95% confidence intervals, respectively. Shaded gray area defines a range of higher TCR binding avidity with a k_off_ threshold set arbitrarily at the slowest off-rate value (lowest k_off_) found for CD8 binding-dependent T-cell clones in **(A)** or **(B)**.

Our results show that Melan-A-specific T-cells bearing CD8 binding-independent TCRs present enhanced binding (Figure 4) and functional avidity (Figure 5) over CD8 binding-dependent T-cells, irrespectively of the vaccine protocol. We therefore evaluated the relationship between TCR-pMHC dissociation rates and functional avidity of CD8 binding-dependent or binding-independent T-cell clones. All clones belonging to a given subgroup but issued from a different vaccine cohort (low peptide/CpG, high CpG, and high peptide) were combined together. In agreement with our recent report (33), we found positive correlations between k_off_-rates and functional avidity as assessed by cytokine production, calcium flux, and cytotoxic activity (Figure 6). Importantly, strong correlations (*P* < 0.01, *r* > 0.5) were only detected for vaccine-induced T-cell clones bearing CD8 binding-dependent TCRs (Figures 6A,B, left panels). By contrast, these correlations were lost when assessing CD8 binding-independent T-cell clones (Figures 6A,B, right panels). This was mostly associated to an increase in frequency of T-cell clones displaying slower off-rates (gray boxes), yet similar functional avidities to many clones of faster off-rates. Statistically significant correlations were recovered, when those high avidity T-cell clones were no longer considered in the analysis (Supplementary Figure 8). Collectively, these findings suggest that above a given TCR off-rate limit, the CD8 binding-independent but not CD8 binding-dependent T-cell clones reach a plateau of maximal functional competence, in terms of functional avidity (i.e., EC_50_).

## Discussion

Immunotherapy against cancer is a rapid and evolving field, currently considered as the fifth pillar of cancer therapy, alongside with surgery, chemotherapy, radiation, and targeted therapy. One of the main challenges of cancer immunotherapy lies in the careful evaluation of tumor-specific T-cell responses offering maximal anti-tumor immune protection and clinical benefit for the patients. In that regard, TCR-pMHC binding avidity (i.e., k_off_ or off-rates) can be viewed as a key T-cell-based biomarker for assessing the functional quality of CD8 T-cells in well-defined clinical settings, such as therapeutic vaccination, helping determine the potency of anti-tumor T-cell responses (34, 37).

In humans, the effect of peptide and adjuvant dosing on both qualitative (i.e., TCR-mediated binding and functional avidity) and kinetic aspects of the CD8 T-cell responses is still not well-established, mainly due to the limitations for the assessment of optimal dosing in clinical trials. In this study, we addressed the question whether the dose of Melan-A peptide and of CpG-B adjuvant used for therapeutic vaccination of melanoma patients can influence the *in vivo* expansion, differentiation, avidity, and function of the responding CD8 T-cells. We show that the use of high peptide dose (0.5 mg) favored the early expansion (i.e., after only 4 vaccine injections) of differentiated effector tumor antigen-specific T-cells bearing TCRs of enhanced binding avidity and low CD8 binding dependency, displaying potent functions. Enrichment of such high avidity CD8 T-cells also occurred in melanoma patients receiving low peptide/CpG (0.1 mg/1–1.3 mg) or high CpG (2.6 mg) doses, but it required additional rounds of vaccination (i.e., 8 vaccine injections) to reach maximal dose response. These observations support the further optimization of cancer vaccines with regard to the antigen dose/density. Actually, designing vaccine regimens with increased peptide dose may allow to accelerate anti-tumor CD8 T-cell responses induced by vaccination, likely important in patients with rapid progressing disease. In addition, vaccines such as peptide/IFA/CpG formulations may be used in combination to other agents, such as immune checkpoint blockade antibodies (49).

Our work offers clear evidence that small changes in peptide concentration (i.e., 5-fold) affected the quality of vaccine-induced CD8 T-cell responses (i.e., TCR binding and functional avidity) early after the start of treatment. In contrast, there was no apparent effect of the CpG adjuvant dose. Consistent with these findings, several studies reported higher rates of antigen-specific T-cell responses when evaluating immunogenicity in high-dose vaccine settings (50–52). This was also shown in a large randomized placebo-controlled trial, in which higher responder rates (i.e., frequency, IFNγ secretion, and proliferation index) were recorded when using higher hepatitis C virus peptide vaccine doses or increasing numbers of vaccinations (53). In a viral setting, vaccines administrated in a dose-escalating fashion stimulated much stronger CD8 T-cell responses and antiviral immunity than a single dose (54). It should be noted that reduced functional avidity with increasing antigen doses was only observed in very few *in vivo* studies using animal models and under particular experimental settings, such as high-dose heterologous peptide during secondary immunization (22) or peptide-pulsed dendritic cell (DC)-based immunization following recall responses (21). Accordingly, the early selection of high avidity CD8 T-cells upon high peptide dose as shown in our study likely illustrates the importance of providing augmented amounts of available specific peptide stimulation for inducing greater immune responses. This effect could be associated with an increase of the number or of the activation state of antigen-presenting DCs. It is also possible that the strength of vaccine-induced CD8 T-cell responses is further improved by synchronizing the number of antigen-presenting cells to the available T-cell precursors (54).

While it is becoming increasingly clear that the antigen dose used for *in vivo* immunization plays a role in modulating vaccine-induced CD8 T-cell responses, a better understanding of the parameters involved in the *in vivo* selection of these T-cells still needs to be determined. In that regard, changes in antigen dose *in vitro* contributes to the shaping of the T-cell repertoire (55). Yet, the outgrowth of a distinct TCR-Vβ subset with low affinity was no longer observed when a high antigen dose was given *in vivo* in peptide-challenged mice (55). Thus, the impact of antigen dose on the TCR repertoire selection *in vivo* might be less significant than *in vitro*, mainly since *in vivo* T-cell activation and function largely rely on the structured engagement of antigen-presenting cells in the immunological environment (56). In line with these observations, individual T-cell clonotypes of high TCR avidity to cognate tumor antigens can be detected for several months up to years after the start of vaccination (8, 20). These data support the notion that once established, the clonal composition of tumor-induced CD8 T-cells, including high avidity clonotypes, can be kept very stable along vaccination. TCR repertoire analysis was not performed in this report, as we decided to focus on the memory-effector EM28^pos^ Melan-A-specific T-cell subset, largely composed of polyclonal TCRαβ clonotypes, with the aim to avoid any bias in avidity associated to the expansion of co-dominant clonotypes, typically found in the EM28^neg^ subset (45). Still, it would be of interest to determine the precise expansion of high vs. low avidity tumor-responding CD8 T-cells over time during vaccination at the clonotype level, but this would require the development of combined single cell TCR-pMHC affinity and sequencing technologies, currently not available.

Besides that TCR-pMHC affinity/avidity plays a pivotal role for T-cell activation and function, CD8 coreceptor also greatly contributes by increasing the sensitivity of antigen recognition upon TCR triggering, by stabilizing TCR-pMHC interactions and promoting TCR/CD3 complex signaling [reviewed in (57)]. The contribution of CD8 to TCR-pMHC interactions during different peptide and CpG dose vaccine settings was further estimated using a CD8 binding-deficient NTAmer variant (37). We found that a significant proportion of vaccine-induced T-cells with lower CD8 binding dependency (i.e., NTAmer-based CD8 binding-independent TCRs) in all three cohorts of patients (i.e., low peptide/CpG, high peptide, and high CpG). However, and consistent with the rapid *in vivo* T-cell expansion and differentiation, a preferential early enrichment (after only 4 vaccine injections) of CD8 binding-independent Melan-A-specific T-cells was observed in patients vaccinated with high peptide dose when compared to those receiving low peptide/CpG or high CpG. Importantly, we demonstrated that CD8 binding-independent T-cells displayed overall superior TCR binding and functional avidities than CD8 binding-dependent ones. Our data are in line with previously documented observations reporting strong anti-viral primary, secondary and memory CD8 T-cell responses in CD8β knock-out mice, favoring the *in vivo* selection of CD8-independent TCRs over CD8αα (58). Moreover, the impact of CD8 on TCR-pMHC binding avidity (48) or function (25) was until recently essentially estimated with CD8 binding proficient vs. deficient pMHC multimers. For instance, Pittet et al. showed that CD8-null multimers allowed the selective identification and isolation of high avidity tumor-reactive CD8 T-cells (39). Here we extended on these observations, by providing the contribution of CD8 on the overall TCR/CD8-pMHC binding avidity during the selection and expansion of responding CD8 T-cell repertoires upon therapeutic peptide vaccination. However, we cannot formally exclude that a significant fraction of tumor antigen-specific CD8 T-cells of lower TCR binding avidity may actually not be stained by pMHC multimer and NTAmer molecules, despite that these reagents display higher sensitivity than Streptamers or Pentamers (33), and may therefore be ignored from our analyses.

Another major finding of this study is that the vaccine-induced CD8 T-cell clones expressing TCRs of increased avidity (i.e., slower TCR off-rates) and CD8 binding-independency reached a plateau of maximal functional competence, corresponding to optimal functional avidity. This, however, could not be observed for the responding CD8 binding-dependent T-cells. Specifically, we show that TCR-ligand avidity correlated well to various aspects of T-cell function (e.g., calcium flux as well as functional avidity for cytokine production and killing capacity), but that this correlation was no longer linear above a certain TCR binding avidity limit. In line with these observations, we reported that virus antigen-specific CD8 T-cells displayed weaker correlations than self/tumor antigen-specific ones, mostly as a consequence of their overall increased TCR binding avidity (33). A recent report further showed that functional avidity of vaccine-responding CD8 T-cells was readily high when combining the strong cationic adjuvant formulation (CAF)09 to low-dose peptide immunization, suggesting that an upper limit for functional avidity had been reached (24). Our findings also nicely fit with other studies indicating that TCR binding affinity/avidity controls the functional profile of cancer-specific CD8 T-cells (29) and defines the optimal balance between effective antitumoral activity and autoimmunity (30). Together, our own and others observations (28–30, 59, 60) are compatible with the well-accepted model proposing that maximal T-cell responsiveness occurs within an optimal window of TCR-pMHC binding interactions, usually lying in the upper physiological TCR affinity range (K_D_ between 10 to 1 μM) [reviewed in (57)]. Moreover, above a given TCR-ligand affinity threshold, antigen-specific CD8 T-cell function cannot be further improved. Notably, tumor-redirected T-cells engineered with increased affinity TCRs (K_D_ < 1 μM) showed substantial defects in T-cell function, which may be limited by negative feedback mechanisms (46, 61).

Collectively, our findings demonstrate that TCR/CD8-pMHC binding avidity is a major determinant of T-cell function, allowing the direct quantification of tumor antigen-specific CD8 T-cell responses following therapeutic peptide vaccination. Specifically, we show that high peptide dose vaccination promoted the early selection of Melan-A-specific T-cells of enhanced TCR binding (i.e., low CD8 binding-dependency) and functional avidity. In contrast, the enrichment of such high-avidity T-cells following low peptide/CpG or high CpG required additional vaccine injections. Measuring the TCR-pMHC binding avidity also provides a reliable, simple-to-use, prone-to-standardization immune metric, while overcoming the major limitations related with T-cell functional assays (33). Importantly, the use of NTAmers permits the precise evaluation of various vaccine protocol combinations (e.g., increasing immunization dose, use of different adjuvants combined to antigen, kinetic of immunization) with the ultimate aim to determine the best vaccine design for clinical efficiency.

## Data Availability Statement

The datasets generated for this study are available on request to the corresponding author.

## Ethics Statement

Study protocols (LUD00-018 and LUD01-003) and groups (III to IV) were designed, approved, and conducted according to the relevant regulatory standards from (i) the Ethics Committee for Clinical Research of the University of Lausanne (Lausanne, Switzerland), (ii) the Protocol Review Committee of the Ludwig Institute for Cancer Research (New York), and (iii) Swissmedic (Bern, Switzerland). Patient recruitment, study procedures, and blood withdrawal were carried out upon written informed consent prior to study inclusion.

## Author Contributions

DS and NR: study design. HM-E: trial coordination. LC-I, BC, PB, and JS: acquisition of data. LC-I, PB, MH, and NR: analysis and interpretation of data. LC-I, DS, MH, and NR: writing and/or revision of the manuscript.

### Conflict of Interest

The authors declare that the research was conducted in the absence of any commercial or financial relationships that could be construed as a potential conflict of interest. The reviewer KL declared a past co-authorship with one of the authors DS to the handling editor.

## Abbreviations

IFA: incomplete Freund's adjuvant
pMHC: peptide presented by MHC
EM: effector-memory
PD-1: programmed cell death 1
NTAmer: NTA-His tag-containing multimer.
