# Supplementary material for: High Peptide Dose Vaccination Promotes the Early Selection of Tumor Antigen-Specific CD8 T-Cells of Enhanced Functional Competence
Source: Front Immunol. 2020 Jan 8;10:3016. doi: 10.3389/fimmu.2019.03016 (PMC6960191; doi:10.3389/fimmu.2019.03016)
Supplement: Supplementary file 1 [file Data_Sheet_1.pdf]

SUPPLEMENTARY MATERIAL

SUPPLEMENTAL MATERIALS AND METHODS

Flow cytometry and cell sorting

For the immune-monitoring analysis (see Figure 1 main manuscript, Suppl. Fig. 1 and Supp. Fig. 2), PBMCs isolated from melanoma patients are regular time-points before and during vaccination, were positively enriched for CD8 T-cells using CD8 Microbeads (Miltenyi) and stained with PE-labeled HLA-A*0201 multimers loaded with analog/ELA Melan-A$^{MART\text{-}1}{}_{26\text{-}35(A27L)}$ (Peptide and Tetramer Core Facility, Ludwig Cancer Research, UNIL CHUV, Lausanne, Switzerland) and the following antibodies: PerCP anti-CD8 (BD Biosciences), ECD anti-CD45RA (Beckman Coulter), APC anti-CD28 and PE-Cy7 anti-CCR7 (BD Biosciences). Samples were acquired using LSRII cytometer (BD Biosciences) and analyzed using FlowJo 9.7.6 software (TreeStar, Inc). For direct *ex vivo* cell sorting and generation of vaccine-induced CD8 T-cell clones (see Suppl. Fig. S2), CD8 T-cells enriched from patient's PBMCs were stained with PE-labeled HLA-A*0201 multimers loaded with analog/ELA Melan-A$^{MART\text{-}1}{}_{26\text{-}35}$ (A27L) (Peptide and Tetramer Core Facility, Ludwig Cancer Research, UNIL CHUV) and the following antibodies: APC-Cy7 anti-CD8, ECD anti-CD45RA (Beckman Coulter), PE-Cy7 anti-CCR7 (Biolegend) and APC anti-CD28 (BD Pharmingen). For surface marker expression analysis (Suppl. Fig S4), vaccine-induced tumor antigen-specific CD8 T-cell clones isolated from the different vaccine cohorts were stained using the following antibodies: BV650 anti-CD69, A700 anti-CD137 (Biolegend), PE anti-CTLA4 (BD Pharmingen), PE-Cy7 anti-TIM-3, APC anti-TIGIT (Invitrogen) and FITC anti-LAG-3 (Enzo).

Functional PD-1 blockade

Vaccine-induced Melan-A-specific CD8 T-cell clones were expanded by stimulation with PHA, IL-2 and feeder cells in the absence (basal) or presence of 20 μg/ml PD-1 blocking antibody (nivolumab; a gift from the Department of Oncology, University Hospital Lausanne, Switzerland). The culture medium containing 20 μg/ml nivolumab was constantly renewed every 3-4 days. After 16-18 days, T-cell clones were assessed for CD107a degranulation and intracellular cytokine production under both culture conditions as in the Patients and Methods section of the main manuscript.

Supplementary Table 1. Clinical characteristics of the vaccinated melanoma patients

Patient	Diagnosis								Vaccination				Outcome			
	Sex	Age	TNM	Breslow	Stage	Stage	Status	Previous treatments	Protocol	Study group	Duration (months)	# of vaccines	Relapse/ Progression	Death	PFS (months)	OS (month)
LAU 205	M	24	pT2aN1bM0	1.40	IIIB	IIIB	NED	Surgery; IFNa adjuvant; immunotherapy (a)	LUD00-018	Group IV (ELA+TYR)	25.2	20	yes	yes	25.1	50.4
LAU 1129	M	52	pT3N0M0	2.50	II	IIIC	NED	Surgery; chemotherapy	LUD00-018	Group IV (ELA+TYR)	9.4	8	yes	yes	9.4	17.0
LAU 1144	M	68	pT3aN0M0	0.60	IIA	IV	NED	Surgery	LUD00-018	Group IV (ELA+TYR)	8.9	8	yes	yes	8.9	29.4
LAU 1164	M	52	pTxNxM1a	-	IV	IV	NED	None	LUD00-018	Group IV (ELA+TYR)	56.6	21			56.6	56.6
LAU 1189	F	68	pT3bN2M0	4.00	IIIB	IIIB	NED	Surgery	LUD00-018	Group IV (ELA+TYR)	3.0	3	yes		10.9	41.7
LAU 1264	M	46	pT3bN0M0	4.00	IIB	IIIB	NED	Surgery; radiotherapy	LUD00-018	Group IV (ELA+TYR)	44.0	26	yes		13.8	125.6
LAU 986	M	30	pT2N1M0	1.15	IIIA	IIIA	NED	Surgery; immunotherapy (a)	LUD01-003	Group IV (EAA+ELA+MAGE+NY)	13.1	12			63.8	63.8
LAU 1286	F	27	pT2aN0M0	1.96	IB	IIIC	NED	Surgery	LUD01-003	Group IV (EAA+ELA+MAGE+NY)	11.1	10			95.8	95.8
LAU 1350	M	57	pT1aN0M0	0.80	IA	IV	ED	Surgery; IFNa adjuvant	LUD01-003	Group IV (EAA+ELA+MAGE+NY)	6.9	6	yes		3.9	34.6
LAU 1352	M	65	pT3bN3M0	3.40	IIIC	IIIC	NED	Surgery	LUD01-003	Group IV (EAA+ELA+MAGE+NY)	13.6	12	yes	yes	14.0	24.5
LAU 616	M	47	pT3N1bM0	2.50	IIIB	IIIC	NED	Surgery; immunotherapy (b); immunotherapy (c)	LUD01-003	Group III (ELA+MAGE+NY)	68.0	36	yes		15.2	146.8
LAU 701	F	70	pT3bN0M0	2.50	IIB	IIIC	NED	Surgery; isolated limb perfusion; immunotherapy (d); immunotherapy (c)	LUD01-003	Group III (ELA+MAGE+NY)	12.6	12			112.4	112.4
LAU 1015	M	75	pT2aN0M1a	1.20	IV	IV	ED	Surgery; immunotherapy (e)	LUD01-003	Group III (ELA+MAGE+NY)	4.2	4	yes	yes	4.2	15.5
LAU 1169	M	61	pT4bN0M0	6.50	IIC	IV	NED	Surgery; isolated limb perfusion	LUD01-003	Group III (ELA+MAGE+NY)	34.8	18	yes		6.7	41.5
LAU 1185	F	57	pT2aN1aM0	1.90	IIIA	IIIC	NED	Surgery; isolated limb perfusion	LUD01-003	Group III (ELA+MAGE+NY)	19.8	13	yes	yes	13.9	42.4

ED: Evidence of disease
NED: No evidence of disease
(a) P40/ELA vaccine protocol: Melan-A ELA peptide + P40 adjuvant
(b) LUDWIG 98-009 vaccine protocol : Melan-A ELA peptide + MAGE-A10 peptide + SB AS-2 adjuvant
(c) LUDWIG 01-003 vaccine protocol: Melan-A ELA peptide + MAGE-A10 peptide + NY-ESO-1b peptide + Montanide ISA-51
(d) LUDWIG 01-0018 vaccine protocol: Melan-A ELA peptide + CpG-7909/ PF-3512676 + Montanide ISA-51
(e) LUDWIG 01-0018 vaccine protocol: Melan-A EAA peptide + Tyrosinase peptide + CpG-7909/ PF-3512676 + Montanide ISA-51
(EAA): Melan-A native peptide; (ELA): Melan-A analog peptide; (TYR): tyrosinase peptide; (MAGE): MAGE-A10; (NY): NY-ESO-1 long peptide
* PFS: progression-free-survival (in months); OS: overall survival (in months)

Supplementary Table 2. Patient characteristics

Patients	15
female (%)	4 (26.7)
male (%)	11 (73.3)
Age at start of protocol	
mean ± std.dev.	57 ± 15
Vaccination duration (months)	
mean ± std.dev.	22.1 ± 19.9
Vaccine number	
mean ± std.dev.	13.9 ± 8.9
Mortality (%)	6 (40)
Progression-free survival (months)	
mean ± std.dev.	30.3 ± 35
Overall survival (months)	
mean ± std.dev.	59.9 ± 41.1

SUPPLEMENTARY FIGURE LEGENDS

Figure S1. *Ex vivo* circulating Melan-A-specific CD8 T-cell frequencies of individual patients following vaccination with increasing peptide or CpG doses. Blood samples of melanoma patients receiving monthly therapeutic vaccinations according to the LUD00-018 (**A**) or LUD01-003 (**B, C**) protocols were analyzed before the start of vaccination (0) and at the indicated vaccine injection (2, 4, 8, 12, 16 or 20). Quantification of Melan-A-specific CD8 T-cell frequencies from patients vaccinated (**A**) with low peptide dose (0.1 mg) and increased CpG-B doses combined to IFA (0.5 mg to 1 mg and to 2 mg), (**B**) with low peptide dose (0.1 mg) and increased CpG-B doses combined to IFA (1.3 mg to 2.6 mg), and **(C)** with high peptide dose (0.5 mg) and low CpG-B dose (1.3 mg) combined to IFA. In contrast to the other patients, patient LAU1015 presented high levels of Melan-A-specific CD8 T-cells with a frequency of 0.35% multimer^{pos} cells already at the start of vaccination. (**A-C**) The dashed line represents the detection threshold (0.01%) of Melan-A-specific CD8 T-cells by *ex vivo* multimer staining. Of note, patients LAU986, LAU1164 and LAU1264 are found in both low peptide/CpG and high CpG cohorts, as they had first been treated in one cohort and after a break in another cohort. (**D**) Multimer^{pos} CD8 T-cells were characterized directly *ex vivo* by flow cytometry for CD28 expression. The proportion of CD28^{pos} Melan-A-specific CD8 T-cells is depicted according to the three vaccine cohorts.

Figure S2. *Ex vivo* characterization of Melan-A-specific CD8 T-cell responses before and after peptide/IFA/CpG vaccination. (**A-C**) Representative dot plots from the seven melanoma patients included in this study and vaccinated with either (**A**) low peptide/CpG, (**B**) high CpG, or (**C**) high peptide dose. Data before vaccination (pre-vacc) and according to the number of received vaccine injections (4v versus 8v) are depicted. The gating strategy (**D**) for the analysis of tumor antigen-specific naive-like versus effector-memory CD8 T-cells is depicted (CD8^{pos}/multimer^{pos} and CD45RA^{pos}/CCR7^{pos} versus CD45RA^{neg}/ CCR7^{neg}). Of note, LAU986 was first treated in the low peptide/CpG cohort (4v), before being included in the high CpG protocol (8v). N/A; not applicable.

Figure S3. Gating strategy for *ex vivo* cell sorting and cloning of Melan-A-specific CD8 T-cells. Following gating on T-cells **(a)**, doublet exclusion **(b)** and living cells **(c)**, we gated on multimer^{pos} or multimer^{neg} CD8^{pos} T-cells **(d)**. On the CD8^{pos}multimer^{neg} gate, the various subset gates were established **(e)**, including naïve (CD45RA^{pos}/CCR7^{pos}), central-memory (CM, CD45RA^{neg}/CCR7^{pos}), effector-memory (EM, CD45RA^{neg}/CCR7^{neg}) and effector-memory CD45RA^{pos} (EMRA, CD45RA^{pos}/CCR7^{neg}). Subset gates were then applied to the CD8^{pos}multimer^{pos} T-cells **(f)**. Using histogram plots, CD28^{pos} and CD28^{neg} gates were established based on the expression of CD28 within total EM multimer^{neg} CD8 T-cells **(g).** This gating was then applied to the EM multimer^{pos} T-cell subset (**h**), allowing the *ex vivo* sorting and subsequent cloning of EM28^{pos} and EM28^{neg} Melan-A-specific CD8 T-cells.

Figure S4. Monomeric TCR/CD8-pMHC dissociation rates of Melan-A-specific CD8 T-cell clones according to the early-differentiated EM28^{pos} phenotype. k_{off} values were obtained following wild-type NTAmer-based dissociation curves of vaccine-induced CD8 T-cell clones and are categorized according to the vaccination cohort (low peptide/CpG dose, gray boxes; high CpG dose, green boxes; high peptide dose, blue boxes) and the number of vaccine injections (4v versus 8v). Data are representative of pooled EM28^{pos} Melan-A-specific T-cell clones (n = 307) and are depicted as box (25th and 75th percentiles) and whisker (10th and

90th percentiles) with the middle line indicating the median. Number of clones *n* and P values by Mann-Whitney *U* test are indicated; * P < 0.05 and *** P < 0.001.

Figure S5. Surface marker expression on CD8 binding-dependent and binding-independent Melan-A-specific CD8 T-cell clones. (A) Quantification of CD69 and CD137 (4-1BB) activation marker expression on CD8 binding-dependent and -independent EM28^{pos} tumor antigen-specific T-cell clones. (**B**) Quantification of the expression of TIM-3, TIGIT, LAG-3 and CTLA4 co-inhibitory receptors on CD8 binding-dependent and -independent EM28^{pos} Melan-A-specific T-cell clones. (**A, B**) Clones were tested under resting or following 24h of stimulation with native Melan-A-specific multimers. Data are depicted as box (25th and 75th percentiles) and whisker (10th and 90th percentiles) with the middle line indicating the median. Number of clones *n* and P values by Mann-Whitney *U* test are indicated; * P < 0.05. Data are representative of 1-4 independent experiments. DEP, CD8 binding-dependent T-cell clones; IND, CD8 binding-independent T-cell clones.

Figure S6. Functional competence of CD8 binding-dependent and -independent Melan-A-specific CD8 T-cell clones according to the vaccine cohort. (**A**) Representative curves from IFNγ and TNFα production assays using graded concentrations of native Melan-A peptide, obtained with T-cells from each vaccine cohort and categorized as CD8 binding-dependent (DEP) or -independent (IND) tumor antigen-specific T-cell clones. The dashed line was arbitrarily set at 10^{-8} M of peptide concentration, allowing direct comparison between CD8 binding-dependent and -independent T-cell clones of each cohort. (**B, C**) EC_{50} and B_{max} values from IFNγ and TNFα production as well as CD107a degranulation performed in 6h co-culture assays using Melan-A-negative T2 target cells pulsed with graded concentration of native Melan-A peptide. Data are representative of EM28^{pos} Melan-A-specific CD8 T-cell clones obtained from the three vaccine cohorts and categorized according to each vaccine cohort and their CD8 binding dependency. Of note, due to the interexperimental variability related to the functional assays, functional avidity (EC_{50}) and maximal response (B_{max}) were measured in separate experiments and thus cannot be compared across the three patient cohorts. Number of clones *n* and P values by Mann-Whitney *U* test are indicated; * P < 0.05 and ** P < 0.01.

Figure S7. Functional competence of Melan-A-specific CD8 T-cell clones in the presence or absence of PD-1 blocking antibody. (**A**) EC_{50} values from IFNγ and TNFα production as well as CD107a degranulation performed in 6h co-culture assays using T2 target cells pulsed with graded concentrations of native Melan-A peptide. (**B**) IFNγ and TNFα production and CD107a degranulation from 6h co-culture assays with T2 target cells loaded with 10^{-5}M of native Melan-A peptide. (**A, B**) CD8 binding-dependent and -independent Melan-A-specific EM28^{pos} T-cell clones were cultured with (+) or without (-) PD-1 blocking mAb nivolumab. P values by Wilcoxon matched-pairs signed rank test; ** P < 0.01, *** P < 0.001 and **** P < 0.0001. (**C**) Quantification of PD-1 expression levels (gMFI) on the CD8 binding-dependent and -independent Melan-A-specific CD8 T-cell clones used for the PD-1 blocking experiment, under resting conditions. DEP, CD8 binding-dependent T-cell clones; IND, CD8 binding-independent T-cell clones. (**A-C**) Of note, PD-1 blockade had an impact on maximal function, but not on functional avidity (EC_{50}). Moreover, increased cytokine production was found for both CD8 binding-dependent and -independent T-cell clones. This may in part be explained by the fact that no major changes in basal PD-1 levels were observed between these selected T-cell clones.

Figure S8. Relationship between TCR dissociation rates and functional avidity of CD8 binding-independent Melan-A-specific CD8 T-cell clones. (**A, B**) Correlations between NTAmer-based TCR dissociation rates (k_{off}, x-axis) and EC_{50} values obtained from CD107a degranulation, IFNγ and TNFα production (y-axis). Each symbol (opened squares) represents an individual vaccine-induced CD8 T-cell clone of high avidity TCRs (i.e. CD8 binding independent). Spearman's correlations (two-tailed, $\alpha = 0.05$) for (**A**) all tested CD8 binding-independent T-cell clones and (**B**) following removal of the highest avidity T-cell clones, present in the shaded grey area. The number of tested clones, the best-fit slope (S) value of the linear regression and the Spearman's correlation coefficient R and P values are indicated. Color-coded and black lines are indicative of regression fitting and 95% confidence intervals, respectively. The shaded grey area defines a range of higher TCR binding avidity with a k_{off} threshold set arbitrarily at the slowest off-rate value (lowest k_{off}) found for CD8 binding-dependent T-cell clones (see Figure 6A, left panels, main manuscript).
