# Supplementary figures and images for: High Peptide Dose Vaccination Promotes the Early Selection of Tumor Antigen-Specific CD8 T-Cells of Enhanced Functional Competence
Source: Front Immunol. 2020 Jan 8;10:3016. doi: 10.3389/fimmu.2019.03016 (PMC6960191; doi:10.3389/fimmu.2019.03016)

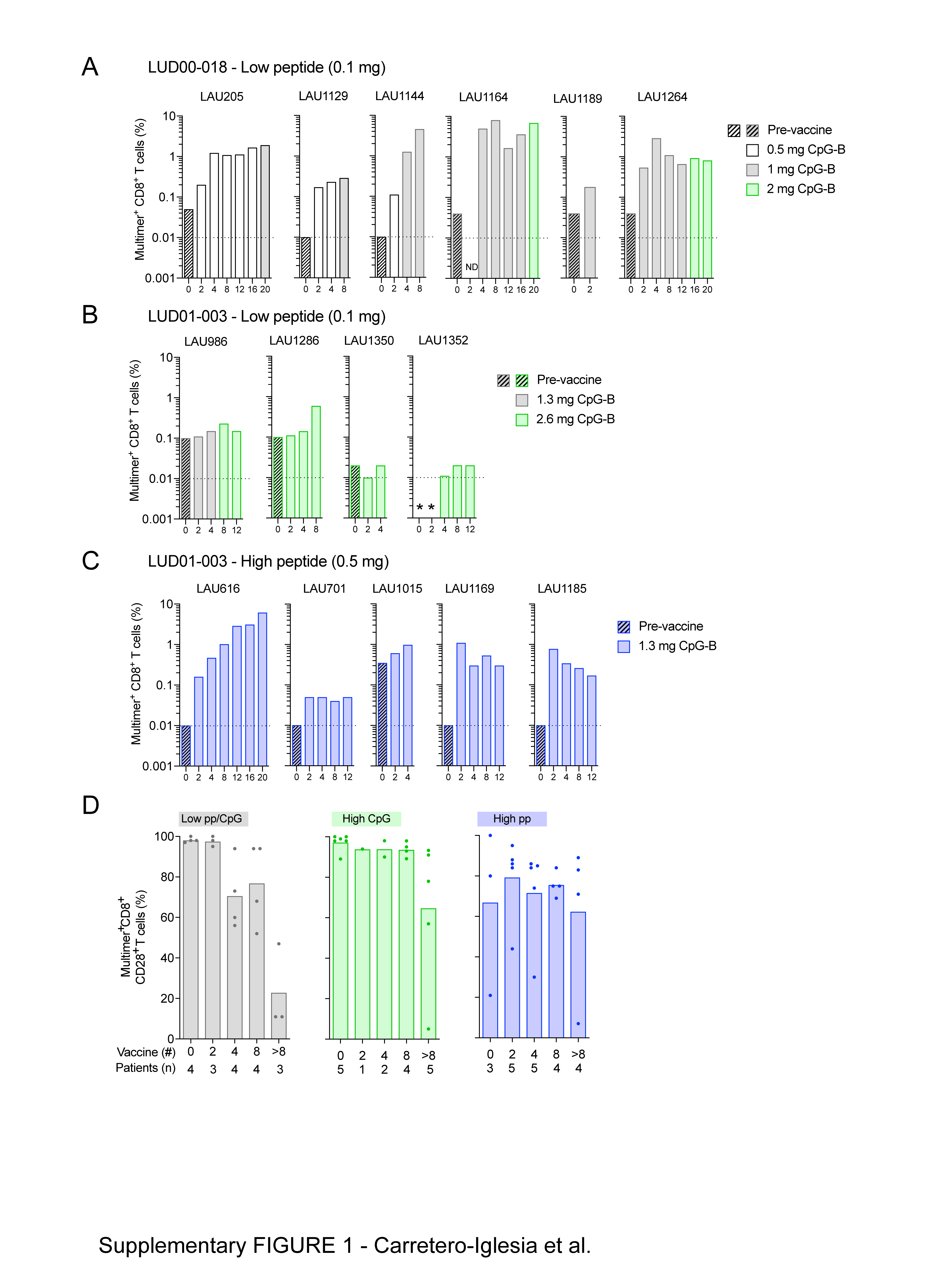

Supplement: Supplementary file 2 [file Image_1.TIF]

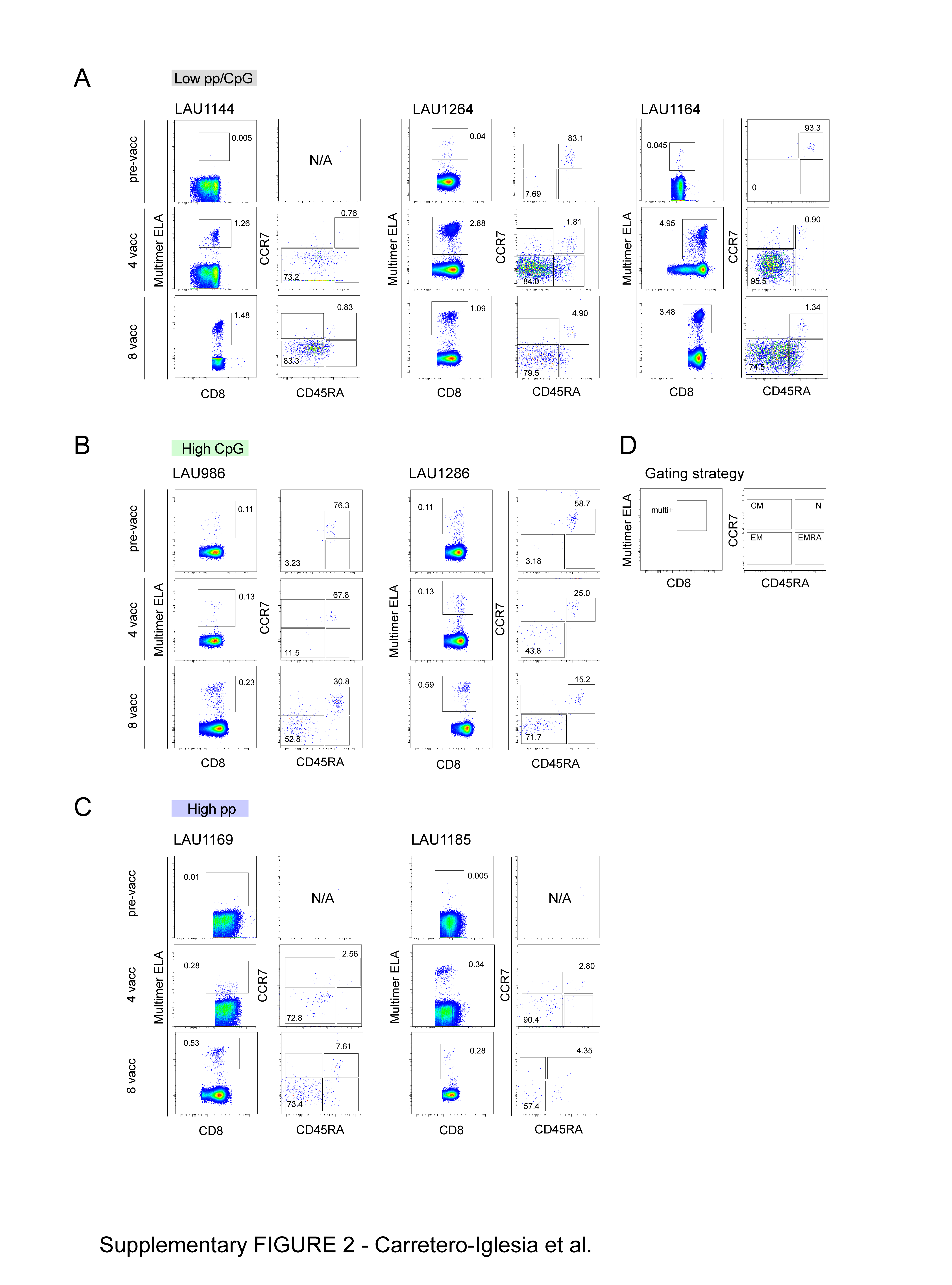

Supplement: Supplementary file 3 [file Image_2.TIF]

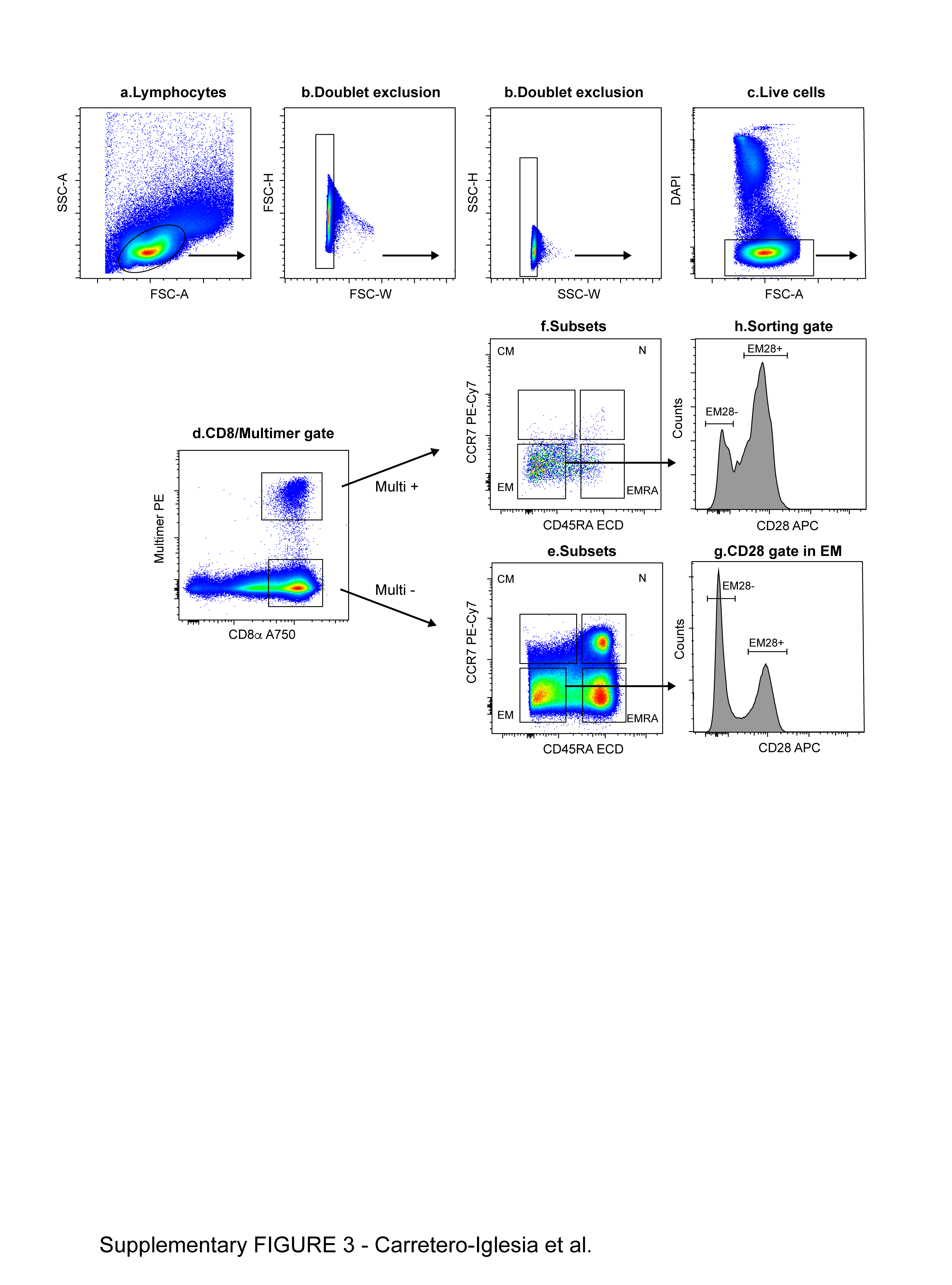

Supplement: Supplementary file 4 [file Image_3.TIF]

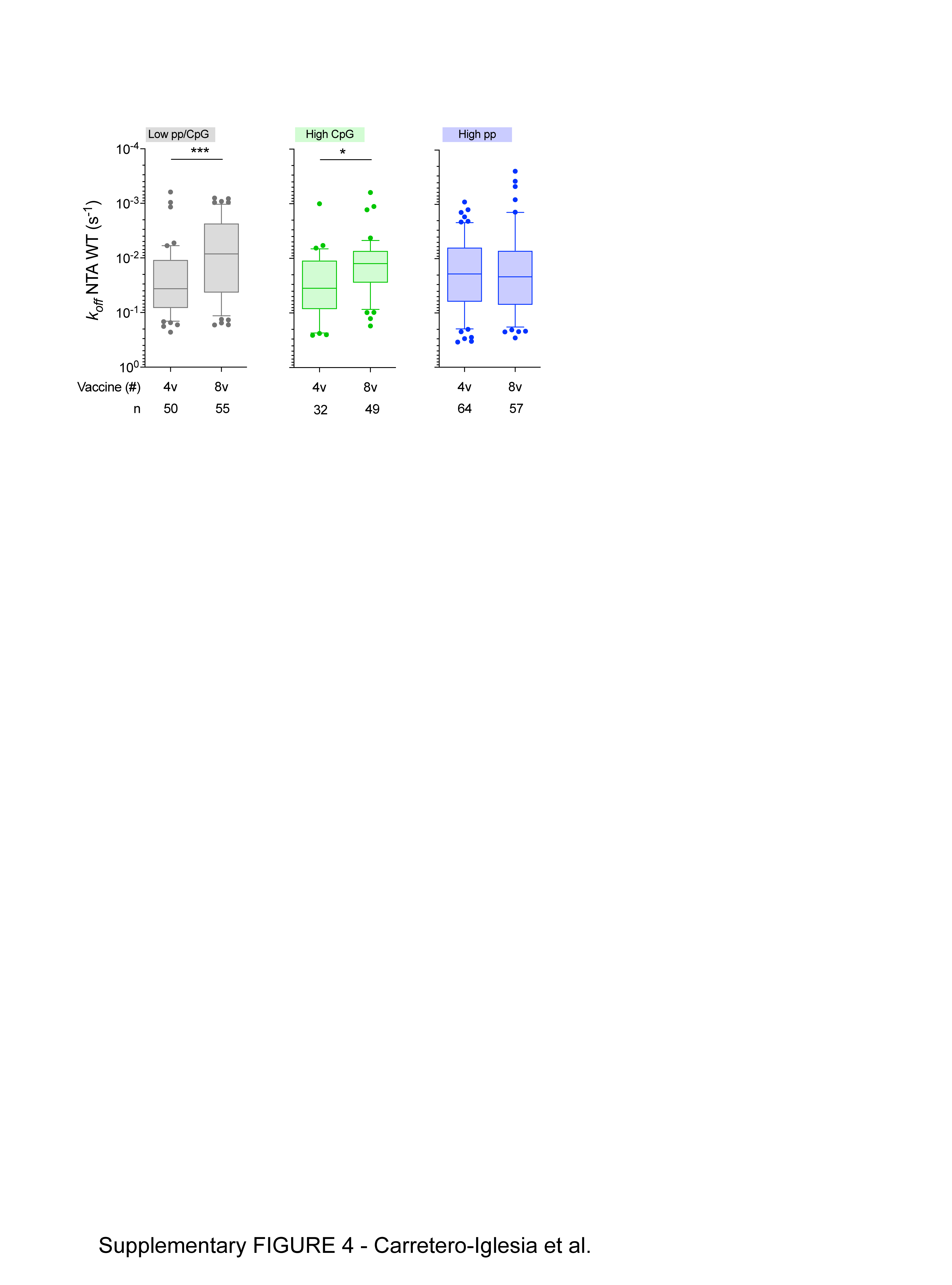

Supplement: Supplementary file 5 [file Image_4.TIF]

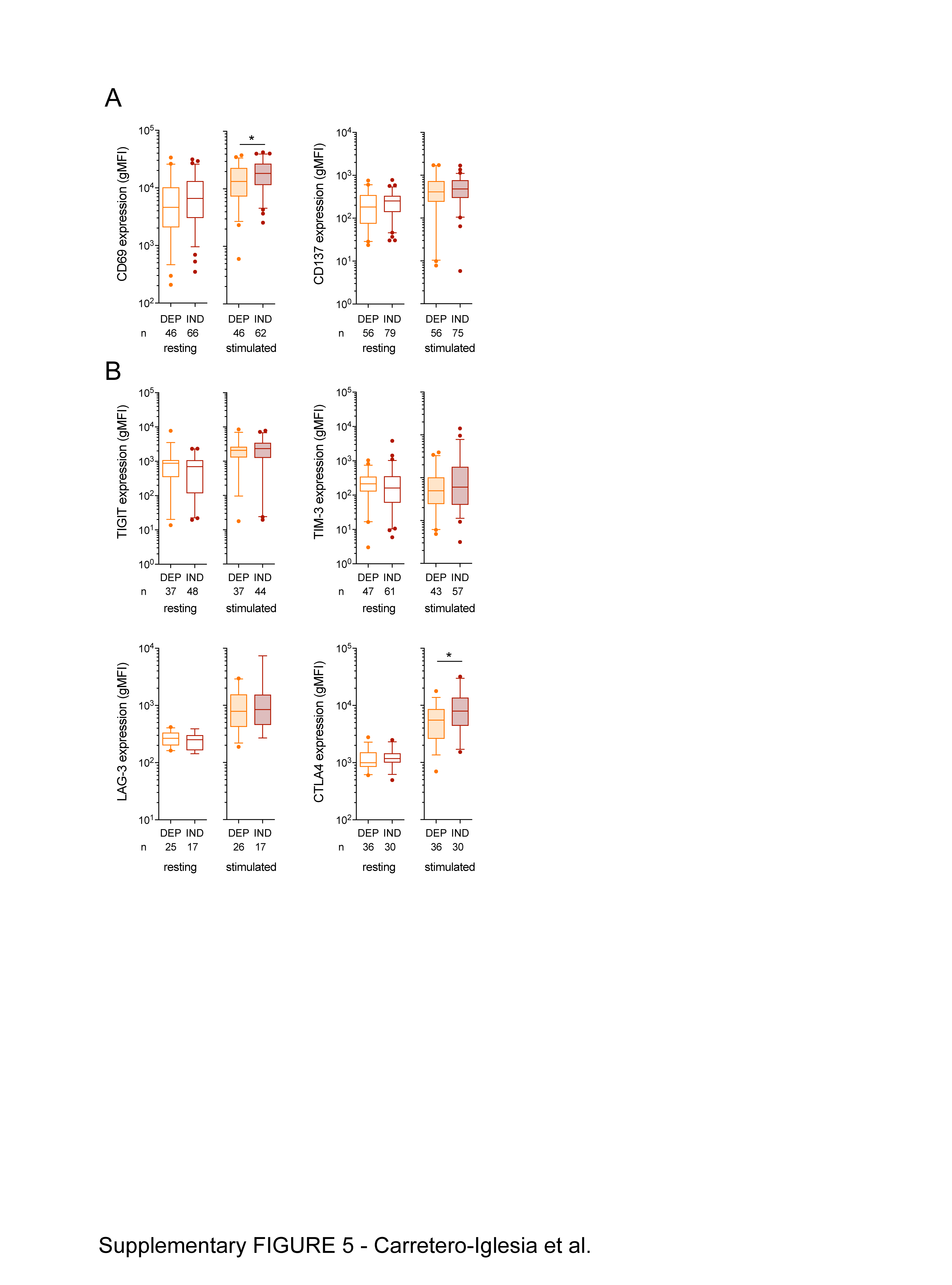

Supplement: Supplementary file 6 [file Image_5.TIF]

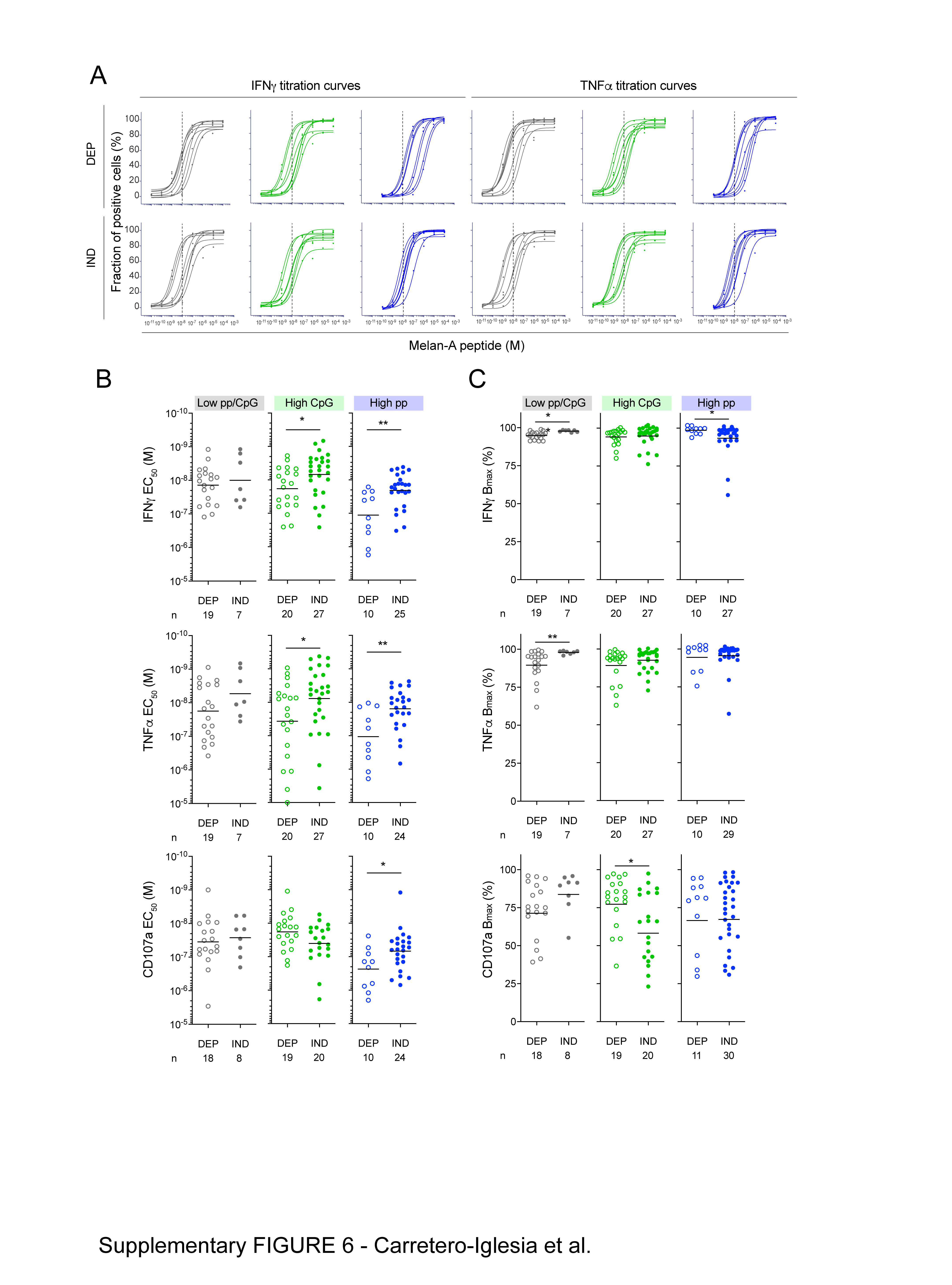

Supplement: Supplementary file 7 [file Image_6.TIF]

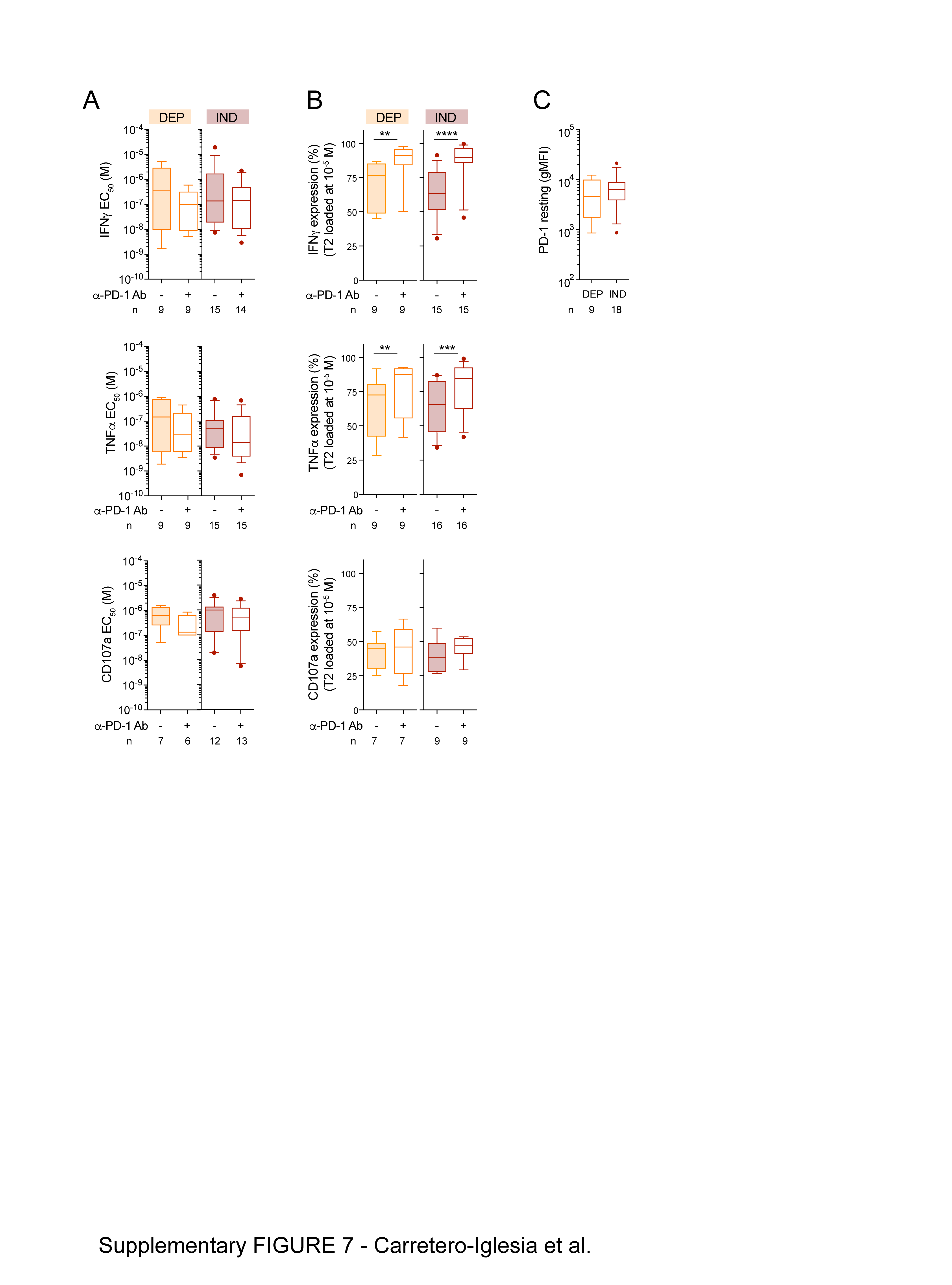

Supplement: Supplementary file 8 [file Image_7.TIF]

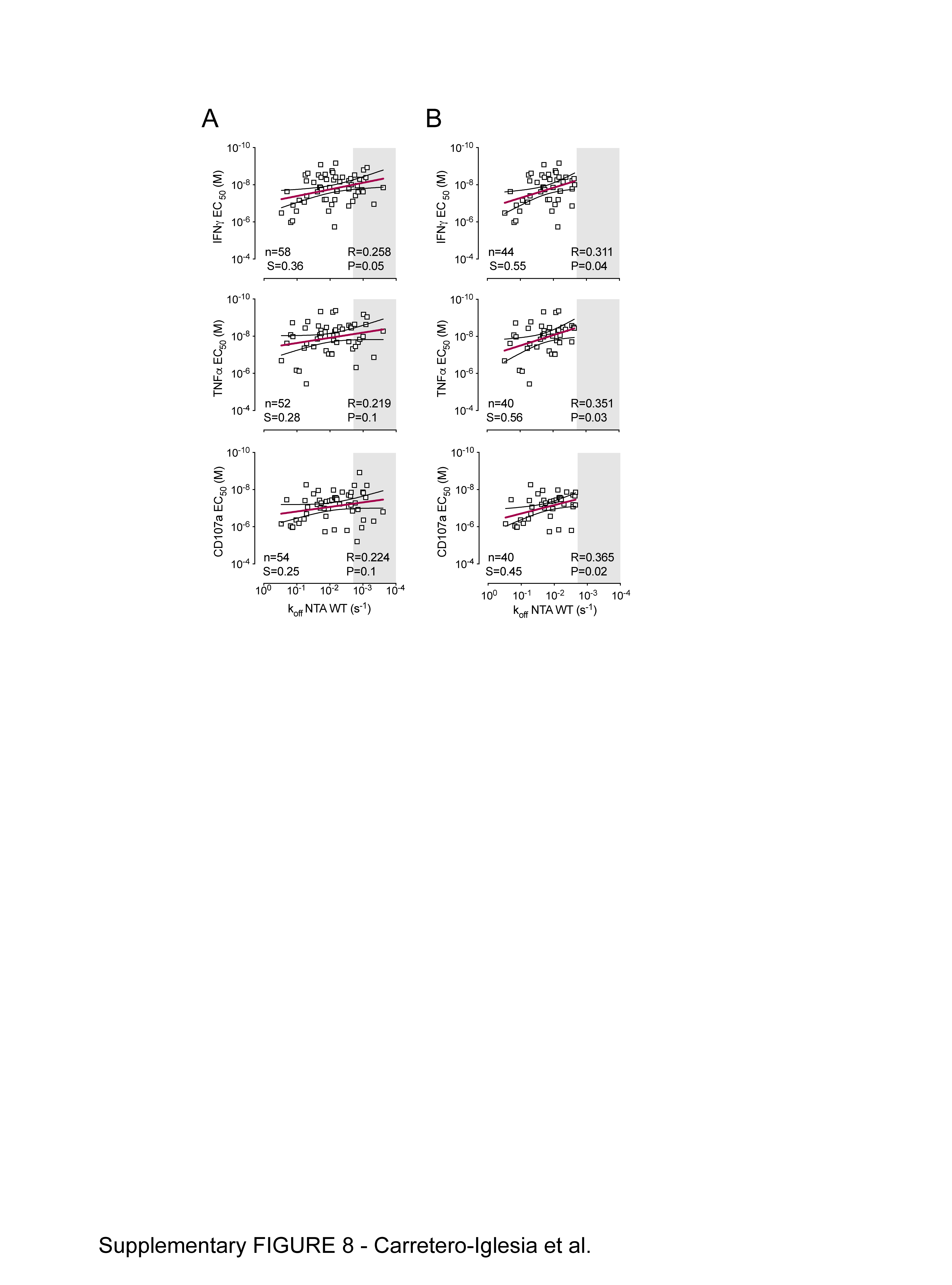

Supplement: Supplementary file 9 [file Image_8.TIF]
